# Molecular Dynamics (MD) Simulations Provide Insights into the Activation Mechanisms of 5-HT_2A_ Receptors

**DOI:** 10.3390/molecules29204935

**Published:** 2024-10-18

**Authors:** Meng Cui, Yongcheng Lu, Mihaly Mezei, Diomedes E. Logothetis

**Affiliations:** 1 Department of Pharmaceutical Sciences, School of Pharmacy, Bouvé College of Health Sciences, Northeastern University, Boston, MA 02115, USA; 2 Center for Drug Discovery, Northeastern University, Boston, MA 02115, USA; 3 Department of Pharmacological Sciences, Icahn School of Medicine at Mount Sinai, New York, NY 10029, USA; mihaly.mezei@mssm.edu; 4 Affiliate of Chemistry and Chemical Biology, Northeastern University, Boston, MA 02115, USA; 5 Affiliate of Bioengineering, Northeastern University, Boston, MA 02115, USA; 6 Affiliate of Roux Institute, Northeastern University, Portland, ME 04101, USA

**Keywords:** G-protein-coupled receptor (GPCR), 5HT_2A_ receptor, MD simulations, protein−ligand interactions, receptor activation, conformational changes

## Abstract

Recent breakthroughs in the determination of atomic resolution 3-D cryo-electron microscopy structures of membrane proteins present an unprecedented opportunity for drug discovery. Structure-based drug discovery utilizing in silico methods enables the study of dynamic connectivity of stable conformations induced by the drug in achieving its effect. With the ever-expanding computational power, simulations of this type reveal protein dynamics in the nano-, micro-, and even millisecond time scales. In the present study, aiming to characterize the protein dynamics of the 5HT_2A_ receptor stimulated by ligands (agonist/antagonist), we performed 1 µs MD simulations on 5HT_2A_/DOI (agonist), 5HT_2A_/GSK215083 (antagonist), and 5HT_2A_ (APO, no ligand) systems. The crystal structure of 5HT_2A_/zotepine (antagonist) (PDB: 6A94) was used to set up the simulation systems in a lipid bilayer environment. We found the monitoring of the ionic lock residue pair (R3.50-E6.30) of 5HT_2A_ in MD simulations to be a good approximation of the effects of agonists (ionic lock breakage) or antagonists (ionic lock formation) on receptor activation. We further performed analyses of the MD trajectories, including Principal Component Analysis (PCA), hydrogen bond, salt bridge, and hydrophobic interaction network analyses, and correlation between residues to identify key elements of receptor activation. Our results suggest that in order to trigger receptor activation, DOI must interact with 5HT_2A_ through residues V5.39, G5.42, S5.43, and S5.46 on TM5, inducing significant conformational changes in the backbone angles of G5.42 and S5.43. DOI also interacted with residues W6.48 (toggle switch) and F6.51 on TM6, causing major conformational shifts in the backbone angles of F6.44 and V6.45. These structural changes were transmitted to the intracellular ends of TM5, TM6, and ICL3, resulting in the breaking of the ionic lock and subsequent G protein activation. The studies could be helpful in future design of selective agonists/antagonists for various serotonin receptors (5HT_1A_, 5HT_2A_, 5HT_2B_, 5HT_2C_, and 5HT_7_) involved in detrimental disorders, such as addiction and schizophrenia.

## 1. Introduction

G-protein-coupled receptors (GPCRs) represent the largest family of membrane proteins, characterized by their seven transmembrane helices. They play a pivotal role in mediating cellular responses to a diverse range of stimuli [1]. GPCRs can be categorized into six distinct families: Class A (rhodopsin-like), Class B (secretin receptor), Class C (metabotropic glutamate receptor), Class D (fungal mating pheromone receptor), Class E (cAMP receptor), and Class F (frizzled/smoothened receptor) [2]. GPCRs are important drug targets, with more than 30% of FDA-approved drugs targeting these receptors [3].

To understand the molecular mechanisms underlying GPCR activation, structural information is essential. In the year 2000, a groundbreaking achievement occurred when the first GPCR structure, that of bovine rhodopsin, was solved in its resting state through X-ray crystallography [4]. Over the past two decades, more than 1000 atomic resolution GPCR structures have been solved (GPCRDB: https://gpcrdb.org/, accessed on 5 August 2024) by advances in crystallography and cryo-electron microscopy (cryo-EM). These structures include both agonist-bound (active) and antagonist-bound (inactive) states, which play a pivotal role in unveiling the molecular basis of GPCR activation. Upon comparing the conformations of GPCRs in their active and inactive states, several critical elements were identified. For example, an outward movement was observed at the intracellular end of TM6, along with the breaking of the ionic lock in opsin (active state) as compared to the rhodopsin structure (inactive state) [5]. In addition to the ionic lock serving as a molecular switch for GPCR activation, other critical switches were identified, such as the toggle switch between W6.48 tryptophan (found in the CWxP motif of TM6) and the Y7.53 tyrosine (located in the NPxxY motif of TM7), which play a pivotal role in the process of GPCR activation [6].

While experimental static structural studies have significantly contributed to our understanding of GPCR function, unraveling the molecular basis for GPCR signaling requires a comprehensive grasp of GPCR dynamics. This encompasses comprehending how a specific GPCR undergoes changes in its shape over time [7]. The increasing computing power has empowered us to explore the dynamic properties of GPCRs, which underlie their functions. This is accomplished through the application of molecular dynamics (MD) simulations. Many MD simulations were carried out on GPCRs to understand their functions and activation mechanisms. A GPCRMD database (https://www.gpcrmd.org/, accessed on 5 August 2024) was created to collect and analyze MD results of GPCRs [8]. Rosenbaum et al. conducted extensive molecular dynamics simulations lasting up to 30 μs on the human β2 adrenergic receptor (β2AR) [9]. Their findings demonstrated that an agonist (BI-167107) bound to the active conformation induces transitions to an inactive-like conformation when not influenced by a G protein or stabilizing antibody [9]. Dror et al. conducted an impressive set of 76 unbiased, all-atom molecular dynamics (MD) simulations of β2AR. These simulations commenced with the active structure, featuring the co-crystallized agonist but excluding the nanobody. The durations of the simulations spanned from 2 to 50 μs. In 36 of these simulations, the receptor spontaneously transitioned from the active conformation to a conformation that closely resembled the inactive structure [10]. Latorraca et al. published an outstanding review on GPCR dynamics [7]. This review comprehensively examines the existing knowledge regarding GPCR dynamics. It delves into the diverse mechanisms through which GPCRs alter their conformation, whether spontaneously or in response to the binding or dissociation of various ligands and intracellular signaling partners. Additionally, the review explores the critical role of receptor dynamics in essential functional processes, including ligand binding, activation, coupling to downstream binding partners such as G proteins and arrestins, biased signaling, and allosteric modulation [7].

While prior protein dynamics studies show promise, we still lack a comprehensive understanding of the intricate dynamic molecular mechanisms underlying GPCR activation. For instance, there remains a gap in our knowledge regarding how conformational changes induced by agonist binding transmit from the binding site to the intracellular end of the receptor and activate G proteins. In this study, we have focused on the serotonin 2A (5HT_2A_) receptor, an extensively studied receptor with a wealth of available experimental structures, including those in agonist-bound and antagonist-bound states [11,12,13,14,15]. Numerous characterized ligands, such as agonists, partial agonists, inverse agonists, and antagonists, have been extensively documented (https://www.guidetopharmacology.org/, accessed on 5 August 2024). Shan et al. conducted MD simulations lasting 350 ns to investigate the conformational changes and dynamics of the 5HT_2A_ receptor induced by various ligands, including the full agonist 5-HT, the partial agonist LSD, and the inverse agonist ketanserin. These simulations were based on a homology model of the 5HT_2A_ receptor. The study revealed distinct effects of these three ligands on well-known key elements of GPCRs, such as the toggle switch W6.48, the ionic lock, and the NPxxY motif in TM7. Furthermore, it unveiled a sequence of steps that connect these essential elements in the process of GPCR activation [16]. Inspired from prior studies, our research aimed to extend this understanding by conducting one-microsecond molecular dynamics (MD) simulations on the experimentally determined 5HT_2A_ receptor structure. We investigated the dynamic behavior in both ligand-bound and ligand-free states of this receptor, with a specific focus on the agonist 2,5-dimethoxy-4-iodoamphetamine (DOI) and antagonist (GSK215803). We were particularly interested in gaining a deeper understanding of the molecular basis of receptor activation induced by the full-agonist 2,5-dimethoxy-4-iodoamphetamine (DOI).

## 2. Results

### 2.1. Agonist-Induced Ionic Lock Broken in 5HT_2A_ during 1 μs MD Simulations

**Figure 1:**
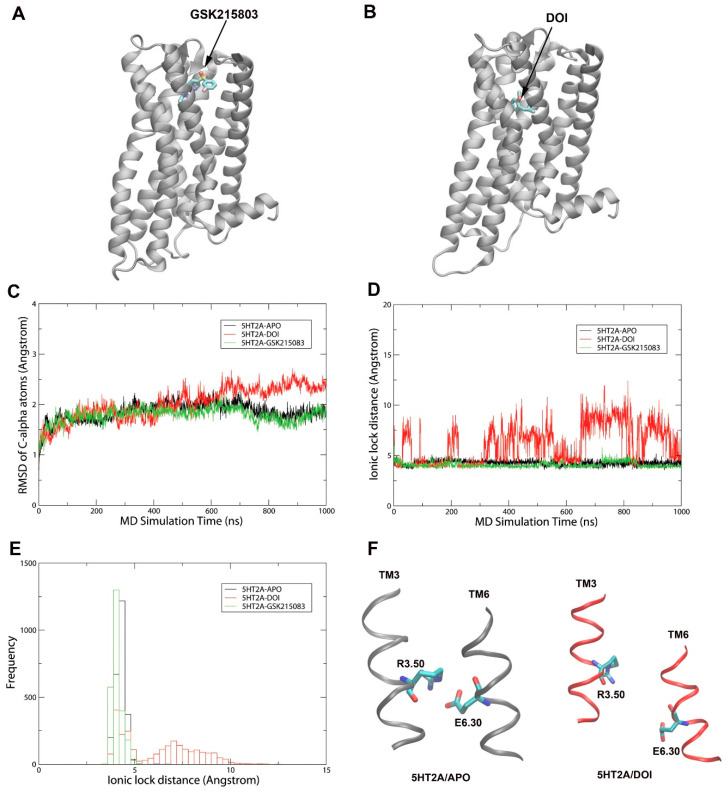
MD simulations on 5HT_2A_−APO, 5HT_2A_−DOI, and 5HT_2A_−GSK215083 for 1 μs. (**A**) Docked 5HT_2A_−DOI; (**B**) 5HT_2A_−GSK215083 complex structures. (**C**) RMSD of Cα atoms of the receptor for the three systems as a function of simulation time (ns). (**D**) Ionic lock distances of the receptors as a function of simulation time (ns). (**E**) Histogram of (**D**,**F**) representative snapshots showing ion lock formed in 5HT_2A_/GSK215803 and broken in 5HT_2A_/DOI.

To understand ligand-induced conformational changes in 5HT_2A_ receptor, we performed 1 µs MD simulations on 5HT_2A_/APO, 5HT_2A_/GSK215803 (antagonist-bound), and 5HT_2A_/DOI (antagonist-bound) states. The structures of the 5HT_2A_/GSK215803 and 5HT_2A_/DOI complexes (Figure 1A,B) were predicted by molecular docking using the AutoDock4.2 program [17]. Figure 1C shows the RMSD of Cα atoms of 5HT_2A_ as a function of the simulation time. The three systems reached equilibrium after 100 ns. The RMSD for the 5TH_2A_/DOI increased between 600 ns and 1 µs simulations, which may indicate agonist-induced conformational changes in receptor activation. Figure 1D,E show the ionic lock distances (R3.50-E6.30) (generic residue numbering) [18] as a function of simulation time. The ionic locks for the 5HT_2A_/APO and 5HT_2A_/GSK215803 systems were formed around 4.5 Å and were stable throughout the 1 µs simulations. In contrast, the ionic lock for the 5HT_2A_/DOI system was broken (ranged from 4.5 to 12.5 Å) for the majority of the simulation time. During the 1 µs MD simulations, the maximum ionic lock distance for the 5HT_2A_/DOI was approximately 12.5 Å, comparable to the experimental active state structure of 5HT_2A_/25-CN-NBOH, which is 14.7 Å [12]. Figure 1F shows representative snapshots of the ionic lock between R3.50 and E6.30 in the 5HT_2A_/APO (ionic lock formed) and 5HT_2A_/DOI (ionic lock broken) systems from MD simulations.

**Figure 2:**
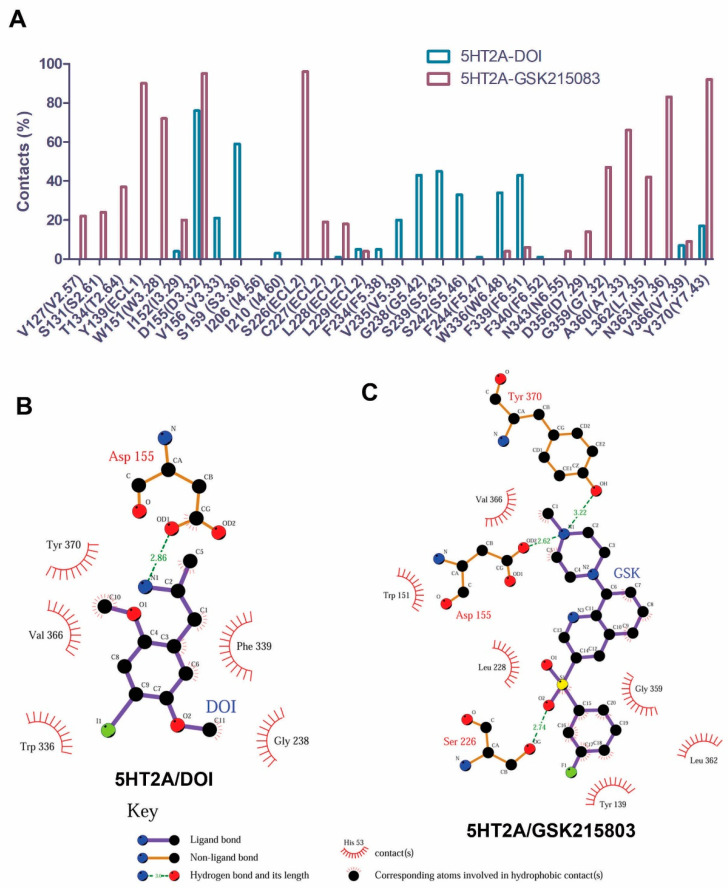
The binding site residues in 5HT_2A_ receptor interact with DOI and GSK215803. (**A**) percentage contacts of the binding site residues in 5HT_2A_ interactions with GSK215803 and DOI during MD simulations (200–1000 ns). (**B**,**C**) 2D ligand−receptor interaction plots for DOI and GSK215803, respectively. Both snapshots were taken from the last frames of the simulations (at 1 µs).

Figure 2A shows percentage contacts of the 5HT_2A_ binding site residues with GSK215803 and DOI during the 1 µs MD simulations. The agonist (DOI) interacted with the residues on TM3 (D3.32, V3.33, and S3.36), TM5 (V5.39, G5.43, S5.44, and S5.46), TM6 (W6.48 and F6.51), and TM7 (V7.39 and Y7.43). While the antagonist (GSK215803) interacted with the residues on the TM2 (V2.57, S2.61, and T2.64), ECL1 (Y139), TM3 (W3.28, I3.29, and V3.33), ECL2 (S226, C227, and L228), TM6 (W6.48, F6.51, and N6.55), and TM7 (D7.29, G7.32, A7.33, L7.35, N7.36, V7.39, and Y7.43). The detailed interactions between DOI/GSK215803 and 5HT_2A_ are shown in Figure 2B,C. Figure S1 shows the tracking of the contact formation between the ligand (DOI or GSK215803) and receptor-binding site residues during the MD simulations.

### 2.2. Agonist-Induced Conformational Changes in 5HT_2A_

**Figure 3:**
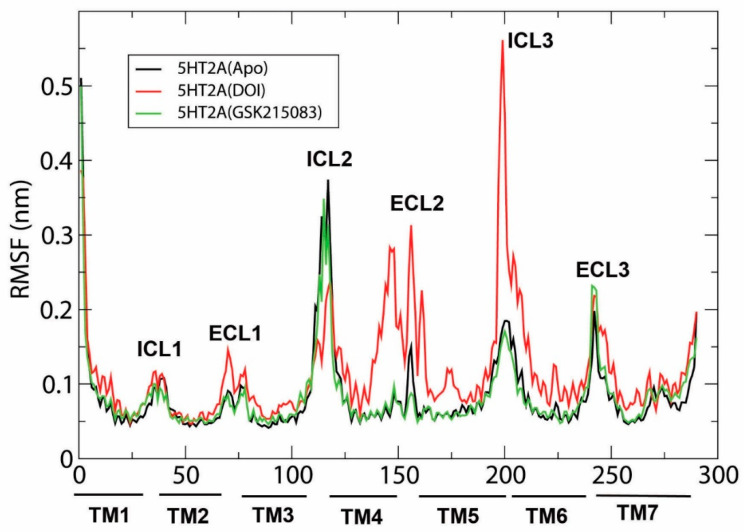
Root mean square fluctuations (RMSF) of the Cα atoms of 5HT_2A_/APO, 5HT_2A_/DOI, and 5HT_2A_/GSK215803 based on the MD simulations (200–1000 ns).

In addition to demonstrating agonist-induced ionic lock breakage, we performed root mean square fluctuation (RMSF) analysis on the three systems (i.e., the 5HT_2A_/APO, 5HT_2A_/DOI, and 5HT_2A_/GSK215803). Figure 3 shows the RMSFs between 5HT_2A_/APO and 5HT_2A_/GSK215803 had very similar profiles. In contrast, the RMSF significantly increased on the extracellular sides of TM4, ECL2, and TM5, as well as on the intracellular sides of TM5, ICL3, and TM6. A slight increase was also observed in ECL1 in the 5HT_2A_/DOI system. The results indicated large conformational changes in these regions caused by DOI and receptor interactions. The large conformational changes in the intracellular end of TM6 may relate to the ion lock broken in the 5HT_2A_/DOI system.

We further analyzed the movements of the TM4 and TM6 during the MD simulations using the Simulaid program [19]. Figure S2 shows projections of the TM helix movements on the X/Y plane (membrane) of either the extracellular or intracellular ends. The TM4 and TM6 in the 5HT_2A_/DOI state underwent a much larger movement on both the extracellular and intracellular sides compared to in the 5HT_2A_/APO state. For example, the movement projections of the intracellular (2.5/3.0 Å) and extracellular (2.5/3.5 Å) ends of TM4 in the 5HT_2A_/APO system increased to 4.5/6.0 Å and 5.0/7.0 Å, respectively, in the 5HT_2A_/DOI system. Similarly, the movement projections of the intracellular (3.0/3.5 Å) and extracellular (3.0/2.5 Å) ends of TM6 in 5HT_2A_/APO increased to 7.0/5.0 Å and 6.0/5.0 Å, respectively, in the 5HT_2A_/DOI system.

**Figure 4:**
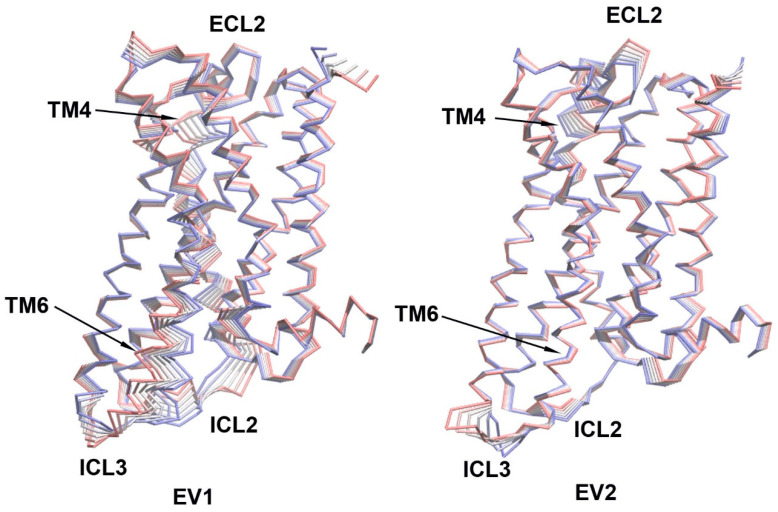
The first and second eigenvectors (EVs) from combined Principal Component Analysis (PCA) of 5HT_2A_−APO/DOI based on the MD simulations (200–1000 ns; Cα atoms of the receptor). The 5HT_2A_ receptor structures were shown as Cα traces (six frames colored from red to blue).

Principal Component Analysis (PCA) can be used to extract the collective motions of the proteins from MD trajectories and also to compare the difference between trajectories of two similar systems, such as apo and holo states (combined PCA) [20,21,22,23]. We performed combined PCA analysis based on the concatenated 5HT_2A_/APO and 5HT_2A_/DOI trajectories (200–1000 ns) using GROMACS (v2020.4). Figure 4 shows the first and second eigenvectors (EVs) from the combined PCA analysis, which contributed 50.35% and 9.28%, respectively, to the overall dynamics of the system. The major conformational differences between the APO and DOI-bound states were in ICL2, TM4, ECL2, ICL3, and TM6, which are consistent with the RMSF results (Figure 3). A 2D projection plot of the first two principal eigenvectors and a plot of eigenvalues versus the corresponding eigenvector indices for the top 20 EVs are given in Figure S3, Table S1, and Movies S1 and S2.

### 2.3. Conformational Changes in the TMs of 5HT_2A_

#### 2.3.1. Prokink

There are three highly conserved proline residues [24] in the TM5, TM6, and TM7 of 5HT_2A_, which kink these helices from their straight conformations. We applied the Prokink protocol (Simulaid program) [19], originally developed by Visiers et al. [25] for numerical evaluation of helix distortions by prolines, to calculate helical bends, wobble angles, and face shifts. In TM5, the average bend, wobble angle, and face shift values for 5HT_2A_/APO were (9.75, 46.85, 63.51), for 5HT_2A_/GSK215803 they were (11.62, 42.16, 59.66), and for 5HT_2A_/DOI they were (9.35, −98.11, 59.09) (Figure S4). Moving to TM6, the respective values were (34.46, −63.11, 89.26) for 5HT_2A_/APO, (34.68, −81.21, 69.45) for 5HT_2A_/GSK215803, and (34.51, −81.31, 56.68) for 5HT_2A_/DOI (Figure S5). Lastly, in TM7, we obtained the following values: 5HT_2A_/APO (28.54, −122.36, 54.55), 5HT_2A_/GSK215803 (28.84, −121.53, 52.41), and 5HT_2A_/DOI (39.90, −68.10, 73.95) (Figure S6). There were no significant changes in the angles calculated by Prokink within TM5 among these systems. However, the variation in wobble angles observed, coupled with the smaller bend angle values, diminished their overall significance (too small bend angle made the definition of the wobble angle meaningless) [25]. In TM6, the face shift value was notably smaller in 5HT_2A_/DOI (56.68) when compared to in 5HT_2A_/APO (89.26) and 5HT_2A_/GSK215803 (69.45). In TM7, the 5HT_2A_/DOI system exhibited an increase in all these values by approximately 11, 54, and 20 degrees, respectively, when compared to the 5HT_2A_/APO and 5HT_2A_/GSK215803 systems, which exhibited similar values.

#### 2.3.2. Tryptophan Toggle Switch W6.48 in the TM6

W6.48 in the TM6 is a highly conserved residue and was proposed to be a critical residue as a toggle switch for family A GPCR activation [26]. We calculated the phi/psi and side chain torsional angle distributions from MD simulation trajectories (Figure S7). The average phi/psi angles for the APO system were (−106.26, −29.79), for the GSK215803-bound system they were (−97.10, −21.52), and for the DOI-bound system they were (−90.82, −15.48). The average side chain torsional angles for the APO system were (−90.06, 113.48), for the GSK215803-bound system they were (−83.91, 102.35), and for the DOI-bound system they were (−79.26, 105.59). The Φ and ψ angles of W6.48 both exhibited increases in the DOI-bound system when compared to in the APO and GSK215803-bound systems. As for the side chain torsional angles, only a minor increase in chi1 was observed in the DOI-bound system when compared to in the APO and GSK215803-bound systems.

#### 2.3.3. Phi/Psi Angle Distributions of Residues F6.44 and V6.45 in the TM6

**Figure 5:**
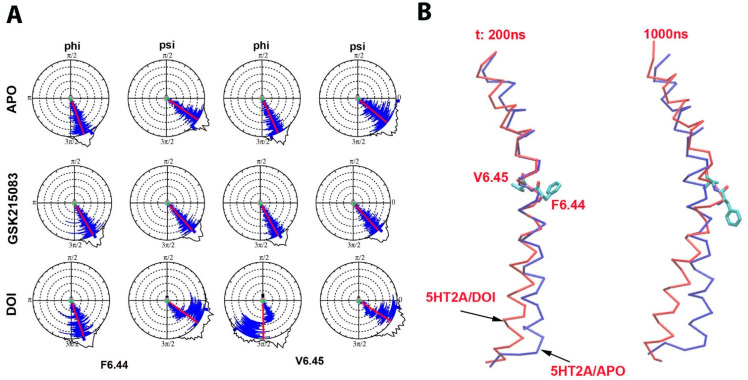
Phi/psi angle distributions of the residues F6.44 and V6.45 in the TM6 for 5HT_2A_/APO, 5HT_2A_/GSK215803, and 5HT_2A_/DOI (200–1000 ns). (**A**) The average phi/psi angles for the APO system were (−69.76/−34.26; −65.33/−39.75), the average phi/psi angles for the GSK215803-bound system were (−59.83/−47.54; −61.38/−47.59), and the average phi/psi angles for the DOI-bound system were (−70.88/−36.11; −89.08/−32.69). (**B**) TM6 conformation comparison between 5HT_2A_/APO (blue) and 5HT_2A_/DOI (red) at 200 ns (left) and 1000 ns (right) during MD simulations.

Since the TM6 underwent large conformational changes in the 5HT_2A_/DOI system during the simulations, we further investigated the phi/psi angle distributions of residues in the helix. Two residues F6.44 and V6.45 were identified with large phi/psi changes in the 5HT_2A_/DOI system during the simulations compared to in the APO and GSK215803-bound systems (Figure 5). The average phi/psi angles of F6.44 and V6.45 for the APO system were (−69.76/−34.26; −65.33/−39.75), for the GSK215803-bound system they were (−59.83/−47.54; −61.38/−47.59), and for the DOI-bound system they were (−70.88/−36.11; −89.08/−32.69). The most significant differences were observed in the average angles for V6.45, where the phi angle decreased but the psi angle increased in the DOI-bound system when compared to in the APO and GSK215803-bound systems. Notably, it is important to highlight that the psi angle of F6.44 and the phi/psi angles of V6.45 underwent substantial changes between the first and second halves of the simulations in the 5HT_2A_/DOI system. These pronounced alterations in backbone torsional angles contributed to the bending of TM6 and an outward shift at the intracellular end of the helix. (Figure 5B and Movie S3). The distributions of side chain torsional angles for residue F6.44 throughout the simulations in the three systems are depicted in Figure S8. Furthermore, significant changes in the side chain torsional angles of F6.44 were observed between the first and second halves of the simulations within the 5HT_2A_/DOI system.

#### 2.3.4. Phi/Psi Angle Distributions of Residues G5.42 and S5.43 in the TM5

The extracellular end of TM5 in 5HT_2A_ underwent substantial conformational changes (as shown in Figure 2) in the presence of DOI, demonstrating increased interactions with DOI compared to GSK215803. To delve into the potential contribution of TM5 to agonist-induced receptor activation, we calculated the phi/psi angle distributions of helix residues during the MD simulations (Figure S9). The average phi/psi angle distributions for residues G5.42 and S5.43 in TM5 for the APO system were (−74.24/26.60; −156.82/−54.86), for the GSK215803-bound system they were (−73.70/25.33; −155.16/−55.72), and for the DOI-bound system they were (−63.15/−42.33; −65.45/−33.30). In the 5HT_2A_/DOI system, significant changes were observed in the psi values of G5.42, with a decrease of approximately 68 degrees, and in the phi values of S5.43, showing an increase of approximately 91 degrees, when compared to in the 5HT_2A_/APO and 5HT_2A_/GSK215803 systems, both of which exhibited similar values.

### 2.4. Salt Bridge Interaction Network Changes in 5HT_2A_ by DOI Activation

**Figure 6:**
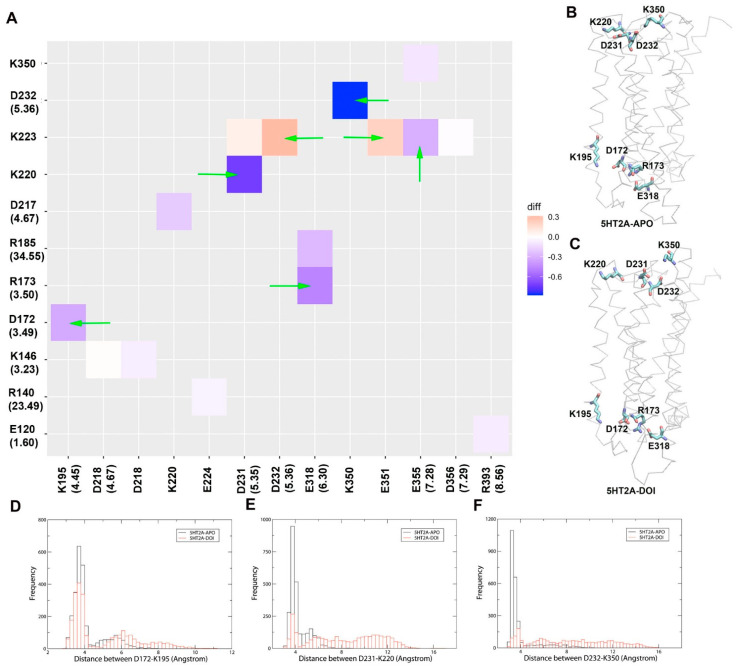
Comparison of key salt bridge interactions between 5HT_2A_−APO and 5HT_2A_−DOI based on MD simulations (200–1000 ns). (**A**) Heatmap plot of salt bridge pairs (red: salt bridge formed; blue: salt bridge disrupted in 5HT_2A_/DOI system). (**B**) The key salt bridge residues in 5HT_2A_−APO and 5HT_2A_−DOI. (**C**–**F**) Histograms of distributions for the distance between D172-K195, D231-K220, and D232-K350, respectively. Green arrows point to the residue pairs shown in (**B**,**C**).

Salt bridge interaction network interactions are crucial for receptor function; for example, the disruption of the ionic lock (R3.50-E6.30) is a key event in GPCR activation [27]. We observed the ionic lock breakage in the 5HT_2A_/DOI system during 1 µs MD simulations (Figure 1). To further study crucial salt bridge interactions involved in receptor activation, we conducted an analysis of the salt bridge interaction network. We quantified the formation of salt bridge pairs as a fraction of the MD trajectories in both the APO and DOI-bound systems and then compared the differences between these two systems. The salt bridge pairs exhibiting the most significant differences in fraction (percentage of salt bridge formation) during the simulations were identified for detailed analysis (refer to Table S2). Figure 6A illustrates these differences in fraction through a heatmap plot, where red squares indicate the formation of salt bridges while blue squares indicate the breaking of salt bridges in the 5HT_2A_/DOI system. Remarkably, our analysis revealed the presence of the ionic lock E318(6.30)-R173(3.50), which exhibited a difference of −0.47 (the negative value means less in fractions of 5HT_2A_/DOI than in 5HT_2A_/APO system) between the DOI-bound and APO systems (Table S2). This discrepancy suggested that the ionic lock was disrupted in the 5HT_2A_/DOI system while remaining stably formed in the 5HT_2A_/APO system. In addition to the ionic lock, several other salt bridges were affected in the 5HT_2A_/DOI system. Notably, the following salt bridges, K350(ECL3)-D232(5.36) (−0.88), D231(5.35)-K220(ECL2) (−0.73), K195(4.45)-D172(3.49) (−0.33), and E355(7.28)-K223(ECL2) (−0.31), were disrupted. Conversely, the 5HT_2A_/DOI system exhibited the formation of new salt bridge pairs, specifically D232(5.36)-K223(ECL2) (0.31) and E351(ECL3)-K223(ECL2) (0.21). Figure 6B,C depict the spatial arrangement of these residues in the 5HT_2A_ receptor bound with DOI and APO, respectively. Figure 6D–F illustrate the distributions of distances for the salt bridge pairs, namely, D172(3.49)-K195(4.45), D231(5.35)-K220(ECL2), and D232(5.36)-K350(ECL3). Figure S10 tracks salt bridge formation within the 5HT_2A_/DOI and 5HT_2A_/APO systems during MD simulations.

### 2.5. Agonist-Induced Hydrogen Bond Interaction Network Changes in 5HT_2A_

**Figure 7:**
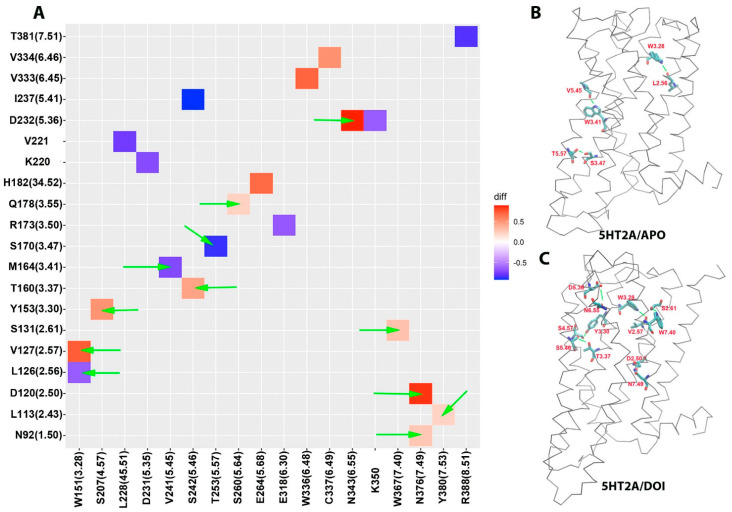
Comparison of hydrogen bond networks between 5HT_2A_/APO and 5HT_2A_/DOI. (**A**) Heatmap plot of hydrogen bond pairs (red: hydrogen bond formed; blue: hydrogen bond disrupted in the 5HT_2A_/DOI system). (**B**) Hydrogen bond formed in the 5HT_2A_/APO system (blue in panel (**A**)). (**C**) Hydrogen bond formed in the 5HT_2A_/DOI system (red in panel (**A**)). Green arrows point to the residue pairs shown in (**B**,**C**).

Hydrogen bond interactions play a crucial role in protein structure and function. For instance, they contribute to the formation of key protein secondary structures like α helices and β sheets. We performed systematically hydrogen bond network analysis based on MD simulation trajectories and compared the hydrogen bond changes in fraction between the 5HT_2A_/DOI and 5HT_2A_/APO systems using the Simulaid program. Figure 7A illustrates the variation in hydrogen bond interaction pairs between the 5HT_2A_/DOI and 5HT_2A_/APO systems using a heatmap plot. In this visualization, red squares indicate an increase in the fraction of hydrogen bond pairs in the 5HT_2A_/DOI system, while blue squares represent a decrease in the fraction of hydrogen bond pairs in the same system. Figure 7B displays a higher fraction of hydrogen bond pairs (T253 (5.57)-S170 (3.47) (−0.87), V241 (5.45)-W164 (3.41) (−0.72), and W151 (3.28)-L126 (2.56) (−0.65)) in the 5HT_2A_/APO system compared to in the 5HT_2A_/DOI system, whereas Figure 7C exhibits a higher fraction of hydrogen bond pairs (N343 (6.55)-D232 (5.36) (0.89), N376 (7.49)-D120 (2.50) (0.85), W151 (3.28)-V127 (2.57) (0.73), S207 (4.57)-153 (3.30) (0.51), S242 (5.46)-T160 (3.37) (0.44), W367 (7.40)-S131 (2.61) (0.30), N376 (7.49)-N92 (1.50) (0.27), S260 (5.64)-Q178 (3.55) (0.22), and Y380 (7.53)-L113 (2.43) (0.21)) in the 5HT_2A_/DOI system compared to in the 5HT_2A_/APO system (Table S3).

### 2.6. Agonist-Induced Hydrophobic Interaction Network Changes in 5HT_2A_

Unlike salt bridges and hydrogen bonds, which rely on electrostatic interactions between polar residues, hydrophobic interactions are driven by the interactions between non-polar residues and water molecules. This drives non-polar residues to associate with each other in energetically favorable states. Hydrophobic interactions play a critical role in protein structure and function. To investigate the significance of hydrophobic residues in 5HT_2A_ receptor activation, we conducted a systematic analysis of hydrophobic interaction networks and calculated the differences in fraction for each hydrophobic residue pair between the 5HT_2A_/DOI and 5HT_2A_/APO systems using the Simulaid program. Figure S11 illustrates the variation in hydrophobic interaction pairs between the 5HT_2A_/DOI and 5HT_2A_/APO systems using a heatmap plot. In this visualization, red squares indicate an increase in the fraction of hydrophobic interaction pairs in the 5HT_2A_/DOI system, while blue squares represent a decrease in the fraction of hydrophobic interaction pairs in the same system. Table S4 presents hydrophobic pairs exhibiting significant differences in fractions between the 5HT_2A_/DOI and APO systems based on MD simulation trajectories.

**Figure 8:**
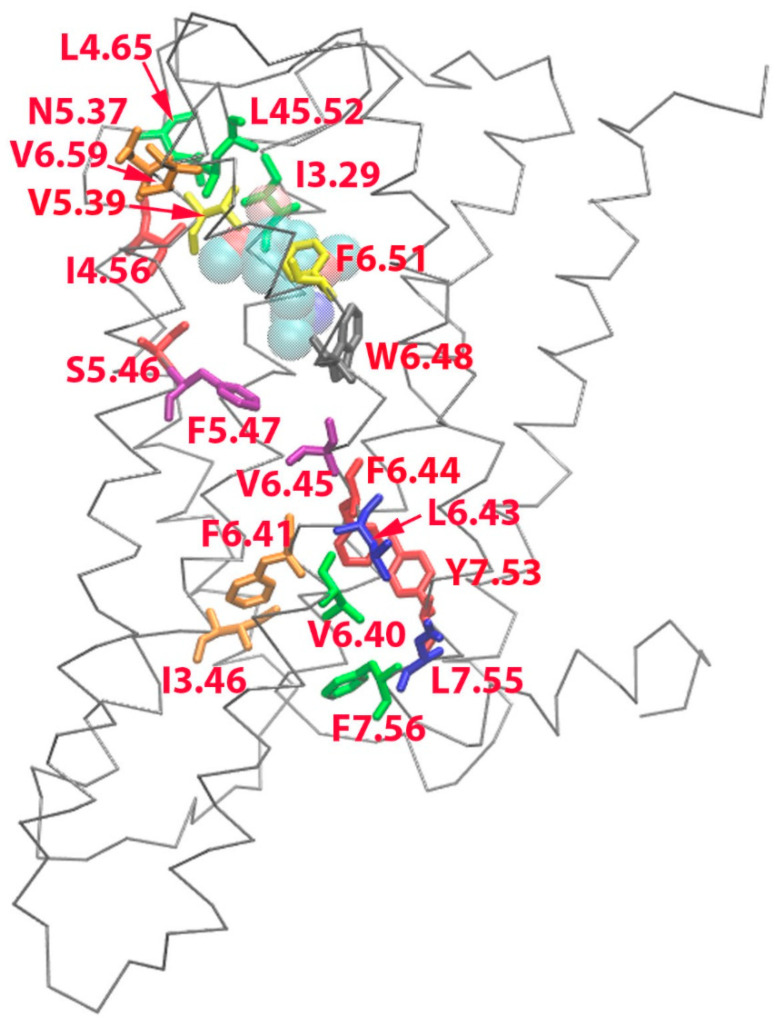
Selected hydrophobic interaction pairs formed in the 5HT_2A_/DOI system but absent in the 5HT_2A_/APO system, which were L45.52-L4.65 (0.46), L45.52-I3.29 (0.45), V6.59-N5.37(0.95), F6.51-V5.39(0.98), S5.46-I4.56 (0.51), V6.45-F5.47(0.46), F6.41-I3.46(0.68), Y7.53-F6.44 (0.44), L7.55-L6.43(0.68), and F7.56-V6.40(0.85). The 5HT_2A_ was drawn in Cα trace, DOI was drawn in vdw spheres, and residues were drawn in sticks.

Figure 8 shows some selected hydrophobic pairs formed in the 5HT_2A_/DOI system but absent in the 5HT_2A_/APO systems. These hydrophobic pairs were L45.52-L4.65 (0.46), L45.52-I3.29 (0.45), V6.59-N5.37(0.95), F6.51-V5.39 (0.98), S5.46-I4.56 (0.51), V6.45-F5.47 (0.46), F6.41-I3.46 (0.68), Y7.53-F6.44 (0.44), L7.55-L6.43 (0.68), and F7.56-V6.40 (0.85). The formation of selected hydrophobic interaction residue pairs in 5HT_2A_/DOI during the simulations is visualized in Movie S4. Notably, the tryptophan residue toggle switch (W6.48) transitioned to a vertical conformation after 250 ns, coinciding with the reorientation of residue F7.56, which shifted its side chain from the outside to the inside of the helix bundle. This conformational change facilitated interactions with V6.40. At 540 ns, residue F6.41 underwent a conformational change, leading to its interaction with I3.46. Subsequently, at 744 ns, residue F6.44 disrupted its interaction with F5.47 and reoriented its side chain toward Y7.53. Additionally, at 704 ns, residues L4.56, L45.52, and I3.29 formed a close spatial arrangement. Notably, residues F6.51, V5.39, L45.52, I3.29, S5.46, I4.56, W6.48, and F5.47 were found to interact with DOI.

### 2.7. Agonist-Induced Residue Pair Correlation Network Changes in 5HT_2A_

The correlation between residue pairs reflects the dynamic characteristics of the protein and can offer valuable insights into receptor activation. We performed a systematic analysis of residue pair correlation and compared the difference in fraction between the 5HT_2A_/DOI and 5HT_2A_/APO systems. Figure S12 illustrates the variation in correlations of residue pairs between the 5HT_2A_/DOI and 5HT_2A_/APO systems using a heatmap plot. In this visualization, red squares indicate an increase in the fraction of correlated residue pairs in the 5HT_2A_/DOI system, while blue squares represent an increase in the fraction of anti-correlation pairs in the same system. Table S5 presents correlated and anti-correlated pairs exhibiting significant differences in fractions between the 5HT_2A_/DOI and APO systems based on MD simulation trajectories.

**Figure 9:**
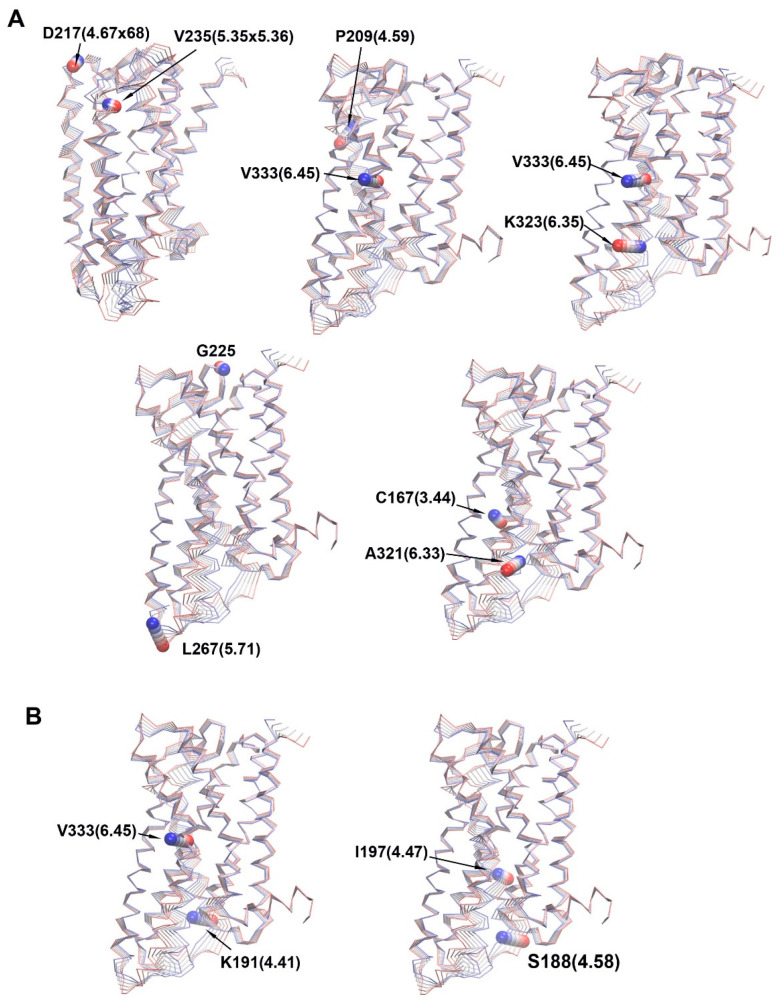
Residue correlation difference between 5HT_2A_/DOI and 5HT_2A_. (**A**) Anti-correlated motions. (**B**) Correlated motions.

Figure 9A displays selected residue pairs exhibiting increased anti-correlation movement in the 5HT_2A_/DOI system, including D217 (4.67 × 68)-V235(5.35 × 36), P209(4.59)-V333(6.45), V333(6.45)-K323(5.35), G225-L267(5.71), and C167(3.44)-A321(6.33). In contrast, Figure 9B showcases selected residue pairs with increased correlation movement in the 5HT_2A_/DOI system, namely, V333 (6.45)-K191(4.41) and I197(4.47)-S188(4.58).

We performed two additional replica simulations for each 5HT_2A_−APO, 5HT_2A_−DOI, and 5HT_2A_−GSK215083 systems. The key elements, such as the ionic lock, crucial for receptor activation, showed consistent trends with the results from the primary MD simulations (Figure S13).

## 3. Discussion

We conducted this study to investigate the molecular basis of 5HT_2A_ receptor activation by DOI using MD simulations. Three systems, namely, 5HT_2A_/APO, 5HT_2A_/GSK215803, and 5HT_2A_/DOI, underwent 1 µs MD simulations, followed by a comprehensive analysis that included PCA, conformational changes, salt bridge, hydrogen bond, hydrophobic, and residue pair correlation network analyses. We found monitoring of the ionic lock residue pair (R3.50-E6.30) of 5HT_2A_ in MD simulations to be a good approximation of the effects of agonists (ionic lock breakage) and antagonists (ionic lock formation) on receptor activation/deactivation, respectively. In the 5HT_2A_/DOI system, the ionic lock was observed to be broken approximately 56% of the time during the simulations (Figure 1D,E). This suggested that the DOI agonist interacted with the receptor, leading to conformational changes necessary for its activation. The RMSF results revealed increased flexibility at the extracellular end of TM4/5 and ECL2 and at the intracellular end of TM5/6, specifically ICL3, when DOI was bound to the receptor in comparison to in the APO and antagonist-bound states (Figure 3). This increased flexibility at the intracellular ends of TM5/6 was in line with the observation of the ionic lock being broken in the DOI-bound state.

The combined PCA results highlighted significant conformational changes occurring in ECL2, TM4, TM6, ICL2, and ICL3 when comparing the 5HT_2A_/APO and 5HT_2A_/DOI systems during the simulations (Figure 4). Intriguingly, our residue pair correlation analysis revealed that the movement of ECL2 was anti-correlated with that of ICL3 in the 5HT_2A_/DOI system (Figure 9). The movement of TM6 played a pivotal role in receptor activation. Notably, we observed a strong anti-correlation between two residues, V6.45 and K6.35, implying that they moved in opposite directions despite being only three alpha turns apart. Additionally, the anti-correlation between residue C3.44, in proximity to R3.50, and residue A6.33, near E6.30, suggests a connection between their motions and the breaking of the ionic lock in the DOI-bound state (Figure 9).

To delve deeper into the key factors driving conformational changes in TM6, we conducted a residue phi/psi angle analysis using Simulaid. This analysis generated dial plots for each phi or psi angle per residue. Our findings revealed significant alterations in the psi angle of F6.44 and both the phi and psi angles of V6.45 in the DOI-bound system compared to in the APO and GSK215803-bound systems (Figure 5A). Remarkably, distinct transitions of these angles were observed between the first and second halves of the simulations. These two residues played a pivotal role in driving conformational changes in TM6, with the angle changes being associated with the outward movement of the intracellular end of TM6 during receptor activation (Figure 5B and Movie S3).

Salt bridge interactions are crucial for receptor activation; for instance, the breaking of the ionic lock is a requisite step in the activation of most family A GPCRs. We conducted a systematic analysis of the salt bridge networks in the 5HT_2A_ receptor in both the APO and DOI-bound states. By comparing the significant differences, we aimed to identify key salt bridge pairs that could play a critical role in receptor activation (Figure 6). Besides the prominent ionic lock (R3.50-E6.30) identified in the analysis, three additional salt bridges, K195(4.45)-D172(3.49), D231(5.35)-K220(ECL2), and K350(ECL3)-D232(5.36), were found to be disrupted in the DOI-bound state in comparison to in the APO state. The salt bridge K195(4.45)-D172(3.49) was in close proximity to the ionic lock, whereas the D231(5.35)-K220(ECL2) and K350(ECL3)-D232(5.36) pairs were situated on the extracellular side of the receptor. To offset the free energy loss associated with the disruption of these salt bridges, new salt bridges, specifically D232(5.36)-K223(ECL2) and E351(ECL3)-K223(ECL2), formed in the DOI-bound states (Table S2).

We conducted a thorough analysis of the hydrogen bond network, revealing notable changes between the APO and DOI-bound states (Figure 7A and Table S3). In the APO state, we identified the presence of hydrogen bonds, specifically T253(5.57)-S170(3.47), V241(5.45)-W164(3.41), and W151(3.28)-L126(2.56) (Figure 7B). However, in the DOI-bound state, these bonds were found to be disrupted. Remarkably, in the DOI-bound state, we observed the emergence of new hydrogen bonds, including N343(6.55)-D232(5.36), N376(7.49)-D120(2.50), W151(3.28)-V127(2.57), and S207(4.57)-153(3.30) (Figure 7C). These newly formed hydrogen bonds played a crucial role in stabilizing the active state of the receptor.

Hydrophobic interaction network analysis aims to identify hydrophobic residue pairs that are closely situated, either through hydrophobic interactions or van der Waals (vdW) interactions. In our study, we conducted a comprehensive comparison of hydrophobic residue pairs between the DOI-bound and APO states of the receptor. Our findings revealed that numerous hydrophobic pairs underwent formation and disruption during the receptor activation process (Figure S11 and Table S4). In Figure 8, we highlighted specific hydrophobic interaction pairs that were formed in the 5HT_2A_/DOI system but were notably absent in the 5HT_2A_/APO systems. During receptor activation, three hydrophobic residues, namely, L4.56, L45.52, and I3.29, located either on or in proximity to ECL2, came into close proximity through hydrophobic interactions. This observation aligned with the significant conformational changes observed in ECL2 as evidenced by RMSF and PCA analyses. It is noteworthy that these hydrophobic residues, along with N5.37, V6.59, V5.39, F6.51, I4.56, and W6.48, collectively constituted a critical part of the DOI-binding site. Furthermore, residues V5.39, W6.48, and F6.51, in conjunction with G5.42, S5.43, and S5.46, acted as pivotal interaction hotspots with DOI (Figure 2). This interaction could be linked to the phi/psi angle alterations observed in G5.42 and S5.43 (Figure S9), which are likely of great significance in the process of receptor activation.

Initially, F6.41 was observed to interact with V5.55 and L5.51. However, upon receptor activation, it underwent a sidechain rotation, directing it to interact with I3.46. This sidechain rotation, in turn, facilitated F6.44 to disengage its interaction with F5.47 and pivot downwards to engage with Y7.53. Subsequently, this flip of sidechains triggered the outward movement of the intracellular end of TM6 and led to the disruption of the ionic lock (Figure 8 and Movie S4).

The phi/psi angles of the tryptophan toggle switch (W6.48) exhibited notable increases in the DOI-bound system during receptor activation (Figure S7). Additionally, the side chain of W6.48 underwent a transition from a horizontal conformation to a vertical one (Movie S4). Prokink analysis revealed that in the DOI-bound state, the proline Face shift value notably decreased in TM6 (Figure S5), whereas the bend, wobble, and face shift angles exhibited an increase in TM7 upon receptor activation (Figure S6). It is worth noting that the wobble and face shift angles clearly exhibited transitions from the first to the second halves of the simulations in the DOI-bound state. These changes may suggest conformational alterations in the NPXXY motif during the receptor activation process.

## 4. Materials and Methods

### 4.1. Molecular Docking

The ligands, DOI and GSK215803, were optimized using an ab initio quantum chemistry method at the HF/6-31G* level, followed by single-point energy calculations of the molecular electrostatic potential for charge fitting using Gaussian 16 (Gaussian, Inc., Wallingford, CT, USA) [28]. The restrained electrostatic potential charge-fitting scheme (RESP) was used to calculate partial charges on the atoms of the ligands [29]. The atomic charges derived from ab initio calculations were used for molecular docking simulations. The crystal structure for 5-HT_2A_R (PDB: 6A94) was processed to add missing sidechains and loops, using Discovery Studio 2017 software (BIOVIA, San Diego, CA, USA). The cytochrome b562 IIG mutant (mbIIG) insertion, which replaced the intracellular loop 3 (ICL3) (residues 266–312) in the crystal structure [11], was removed and regenerated with a short loop instead to connect the intracellular ends of TM5 and TM6. AutoDock 4.2 [30] was used to dock molecules into the receptors with selected sidechain flexible residues in the binding pocket. A grid map was generated for the receptor using C, H, N, O, S, F, and I (i.e., carbon, hydrogen, nitrogen, oxygen, sulfur, fluorine, and iodine) elements sampled on a uniform grid containing 80 × 80 × 80 points, with each point 0.375 Å apart. The Lamarckian genetic algorithm was selected to generate ligand-binding conformations. For each ligand, 100 docking simulations were performed. The final docked ligand conformations were selected based on binding energies and cluster analysis.

### 4.2. MD Simulations

Protonation states of the titratable residues in 5-HT_2A_ receptors were calculated at pH = 7.4 using the H++ server (http://biophysics.cs.vt.edu/, accessed on 22 May 2023) [31]. The ligand−receptor complexes identified in the molecular docks were inserted into a simulated lipid bilayer composed of POPC:POPE:cholesterol (2:2:1) [32] and a water box using the CHARMM-GUI Membrane Builder webserver (http://www.charmm-gui.org, accessed on 22 May 2023) [33]. Sodium chloride (150 mM) as well as neutralizing counter ions were applied to the systems. The total atom numbers were 68,150, 68,179, and 68,195 for the 5HT_2A_/APO, 5HT_2A_/DOI, and 5HT_2A_/GSK215803, respectively. The PMEMD.CUDA program of AMBER 20 was used to conduct MD simulations [34]. The Amber ff14SB, lipid17, and TIP3P force fields were used for the receptors, lipids, and water. The parameters of DOI and GSK215803 were generated using the general AMBER force field (GAFF) by the Antechamber module of AmberTools 17, using the partial charge determined via RESP by ab initio quantum chemistry at the HF/6-31G* level [29]. Coordinate files and system topology were established using the tle ap module of Amber. The systems were energetically minimized by 500 steps (with a position restraint of 500 kcal/mol/Å^2^) followed by 2000 steps (without a position restraint) using the steepest descent algorithm. Heat was then applied to the systems to drive the temperature from 0 to 303 K using Langevin dynamics with a collision frequency of 1 ps^−1^. Receptor complexes were position-restrained using an initial constant force of 500 kcal/mol/Å^2^ during the heating process, which was subsequently diminished to 10 kcal/mol/Å^2^, allowing the lipid and water molecules free movement. Before the MD simulations, the systems underwent a 5 ns equilibration. Then, a total of 1000 ns of MD simulations were conducted using hydrogen mass repartitioning and a time step of 4 fs. The coordinates were saved every 100 ps for analysis. The simulations were conducted in an isothermal and isobaric nature, with the pressure maintained using an isotropic position scaling algorithm with the pressure relaxation time fixed at 2 ps. Long-range electrostatics were calculated by the particle mesh Ewald method with a 10 Å cut-off [35]. Three replica simulations for each of the 5HT_2A_−APO, 5HT_2A_−DOI, and 5HT_2A_−GSK215083 systems were performed. The results of the MD simulations were analyzed using various tools and methods, including the built-in utilities of the GROMACS program from Groningen University (Groningen, The Netherlands), Simulaid (https://mezeim01.dmz.hpc.mssm.edu/simulaid/, accessed on 22 May 2023) [19], as well as in-house scripts. For the residue labeling in the 5HT_2A_ receptor, the classical (Ballesteros–Weinstein) numbering scheme [18] was employed.

## 5. Conclusions

By using MD simulations, we investigated the molecular basis for the 5HT_2A_ activation process by the agonist, DOI. The ionic lock (R3.50-E6.30) breakage was observed during the 1 µs MD simulation in the 5HT_2A_/DOI system. Multiple approach analysis, including RMSF and combined PCA, showed that DOI induced large conformational changes at the extracellular end of TM4/5 and ECL2, as well as at the intracellular end of TM5/6, specifically the ICL3 of the receptor. The large conformational changes in the TM6 were caused by a toggle switch (W6.48) which triggered phi/psi angle changes in the residue F6.44 and V6.45. By comparing the salt bridge, hydrogen bond, hydrophobic interaction networks, and residue pair correlations, we identified some key elements for the receptor activation. Our results suggest that in order to trigger receptor activation, DOI must interact with 5HT_2A_ through residues V5.39, G5.42, S5.43, and S5.46 on TM5, inducing significant conformational changes in the backbone angles of G5.42 and S5.43. DOI also interacted with residues W6.48 (toggle switch) and F6.51 on TM6, causing major conformational shifts in the backbone angles of F6.44 and V6.45. These structural changes were transmitted to the intracellular ends of TM5, TM6, and ICL3, resulting in the breaking of the ionic lock and subsequent G protein activation. The studies could be useful for designing selective agonists/antagonists for various serotonin receptors (5HT_1A_, 5HT_2A_, 5HT_2B_, 5HT_2C_, and 5HT_7_) involved in detrimental disorders such as addiction and schizophrenia in the future.

## Data Availability

Data are contained within the article and Supplementary Materials.

## Supplementary Materials

The following supporting information can be downloaded at: https://www.mdpi.com/article/10.3390/molecules29204935/s1, Figure S1: Selected contact tracking during the MD simulations (200–1000 ns) of 5HT_2A_/DOI and 5HT_2A_/GSK215803; Figure S2: The projection of TM4 (K4.41-Q4.66) and TM6 (S6.25-C6.49) in 5HT_2A_ movement along the membrane plan during 1 µs simulations (200–1000 ns); Figure S3: Eigenvectors (EVs) from Combined Principal Component Analysis (PCA) of 5HT_2A_-APO/DOI based on the MD simulations (200–1000 ns, Cα atoms of the receptor); Figure S4: Dial plots for Proline Kink P246 (5.50) in TM5 for 5HT_2A_/APO, 5HT_2A_/GSK215803 and 5HT_2A_/DOI (200–1000 ns); Figure S5: Dial plots for Proline Kink P338 (6.50) in TM6 for 5HT_2A_/APO, 5HT_2A_/GSK215803 and 5HT_2A_/DOI (200–1000 ns); Figure S6: Dial plots for Proline Kink P377 (7.50) in TM7 for 5HT_2A_/APO, 5HT_2A_/GSK215803 and 5HT_2A_/DOI (200–1000 ns); Figure S7: Dial plots for the Tryptophan Toggle Switch W6.48 in the TM6 for 5HT_2A_/APO, 5HT_2A_/GSK215803 and 5HT_2A_/DOI (200–1000 ns); Figure S8: Dial plots for side chain torsional angles of F6.44 in the TM6 for 5HT_2A_/APO, 5HT_2A_/GSK215803 and 5HT_2A_/DOI (200–1000 ns); Figure S9: Dial plots of phi/psi angle distributions for residues G5.42 and S5.43 in the TM5 for 5HT_2A_/APO, 5HT_2A_/GSK215803 and 5HT_2A_/DOI (200–1000 ns); Figure S10: Selected salt bridge tracking during the MD simulations (200–1000 ns) of 5HT_2A_/DOI and 5HT_2A_/APO; Figure S11: Heat maps for hydrophobic interaction difference between 5HT_2A_/APO and 5HT_2A_/DOI during MD simulations (200–1000 ns); Figure S12: Heat maps for correlation pair difference between 5HT_2A_/APO and 5HT_2A_/DOI during MD simulations (200–1000 ns); Figure S13: Ionic lock distances of the receptors as function of simulation time (ns) for three replica; Table S1: The eigenvalues and percentage contribution of each eigenvector (top 20); Table S2: Formed and broken salt bridge pairs in the 5HT_2A_/DOI system compared to the APO system from MD simulation trajectories (200–1000 ns); Table S3: Formed and broken hydrogen bond pairs in the 5HT_2A_/DOI system compared to the APO system from MD simulation trajectories (200–1000 ns); Table S4: Formed and broken hydrophobic interaction pairs in the 5HT_2A_/DOI system compared to the APO system from MD simulation trajectories (200–1000 ns); Table S5: Anti-correlation and correlation pairs in the 5HT_2A_/DOI system compared to the APO system from MD simulation trajectories (200–1000 ns); Movie S1: First eigenvector (EV1) from combined PCA analysis based on the concatenated 5HT_2A_/APO and 5HT_2A_/DOI trajectories (200–1000 ns); Movie S2: Second eigenvector (EV2) from combined PCA analysis based on the concatenated 5HT_2A_/APO and 5HT_2A_/DOI trajectories (200–1000 ns); Movie S3: The bending of TM6 and an outward shift at the intracellular end of the helix in 5HT_2A_/DOI during MD simulations; Movie S4: The formation of selected hydrophobic interaction residue pairs in 5HT_2A_/DOI during the simulations.

## Abbreviations

MD, molecular dynamics; DOI, 2,5-dimethoxy-4-iodoamphetamine; PCA, Principal Component Analysis; GPCR, G-protein-coupled receptor; cryo-EM, cryo-electron microscopy; RMSF, root mean square fluctuation; ICL, intracellular loop; ECL, extracellular loop; TM, transmembrane.
