# Supplementary material for: Molecular Dynamics (MD) Simulations Provide Insights into the Activation Mechanisms of 5-HT2A Receptors
Source: Molecules. 2024 Oct 18;29(20):4935. doi: 10.3390/molecules29204935 (PMC11510212; doi:10.3390/molecules29204935)

Supplementary materials

Molecular Dynamics (MD) Simulations Provide Insights into the Activation Mechanisms of 5-HT$_{2A}$ Receptors

Meng Cui [1,2,*], Yongcheng Lu [1], Mihaly Mezei [3], Diomedes E. Logothetis [1,2,4,5,6,*]

1 Department of Pharmaceutical Sciences, School of Pharmacy, Bouvé College of Health Sciences,

Northeastern University, Boston, MA 02115, USA

2 Center for Drug Discovery, Northeastern University, Boston, MA 02115, USA

3 Department of Pharmacological Sciences, Icahn School of Medicine at Mount Sinai, New York, NY 10029, USA; mihaly.mezei@mssm.edu

4 Affiliate of Chemistry and Chemical Biology, Northeastern University, Boston, MA 02115, USA

5 Affiliate of Bioengineering, Northeastern University, Boston, MA 02115, USA

6 Affiliate of Roux Institute, Northeastern University, Portland, ME 04101, USA

* Correspondence: m.cui@northeastern.edu (M.C.); d.logothetis@northeastern.edu (D.E.L.)

Figure S1. Selected contact tracking during the MD simulations (200-1000ns) of 5HT$_{2A}$/DOI (A) and 5HT$_{2A}$/GSK215803 (B).

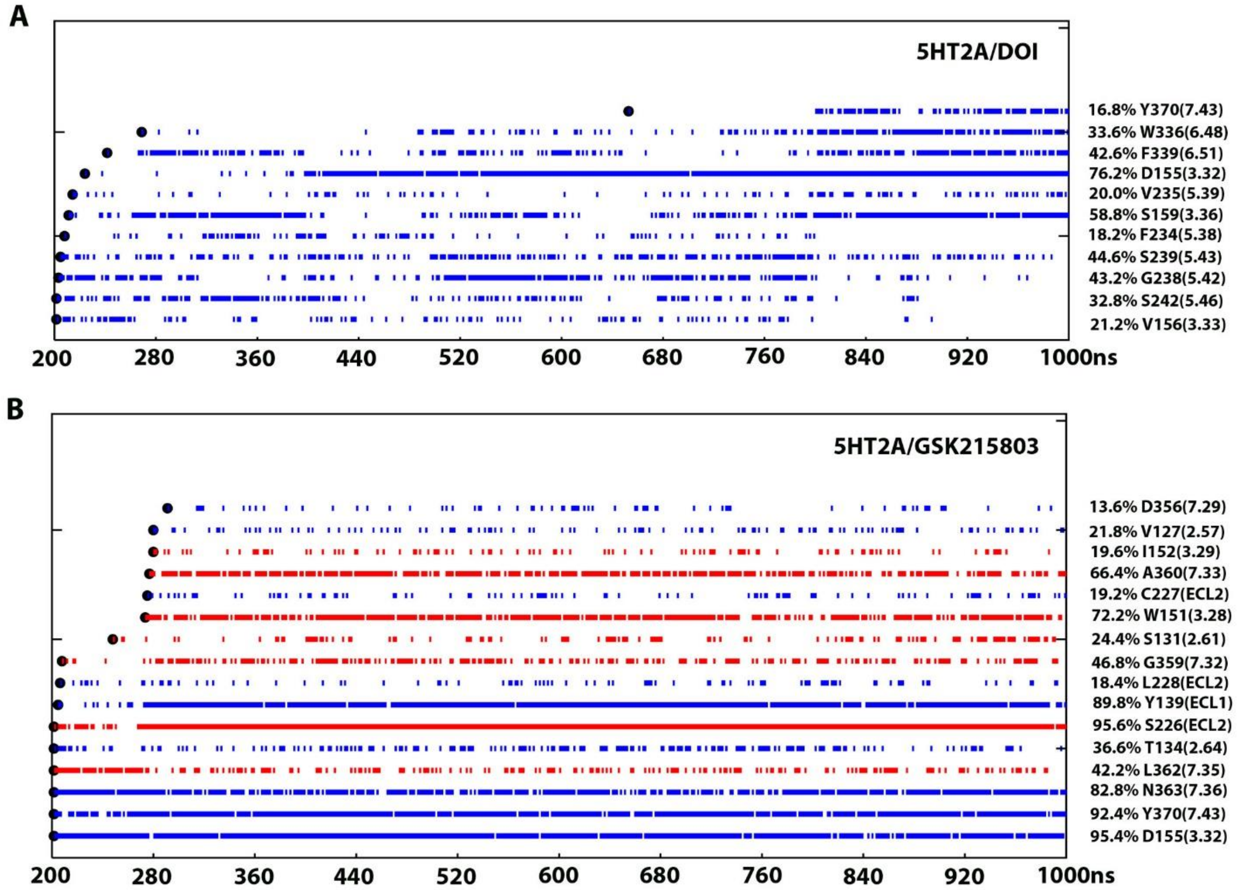

Figure S2. The projection of TM4 (K4.41-Q4.66) and TM6 (S6.25-C6.49) in 5HT$_{2A}$ movement along the membrane plan during 1μs simulations (200 -1000ns).

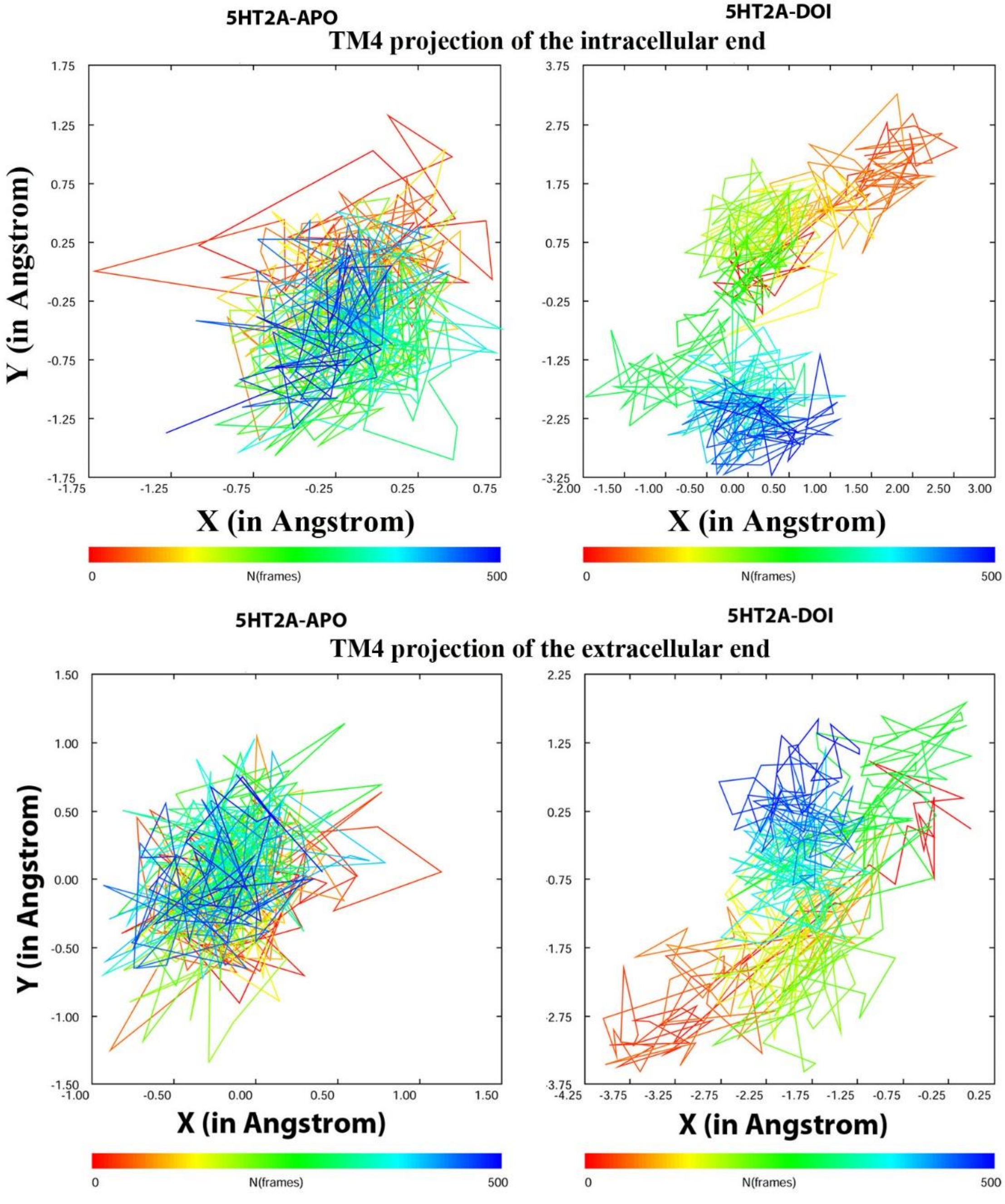

Figure S2 (Continue)

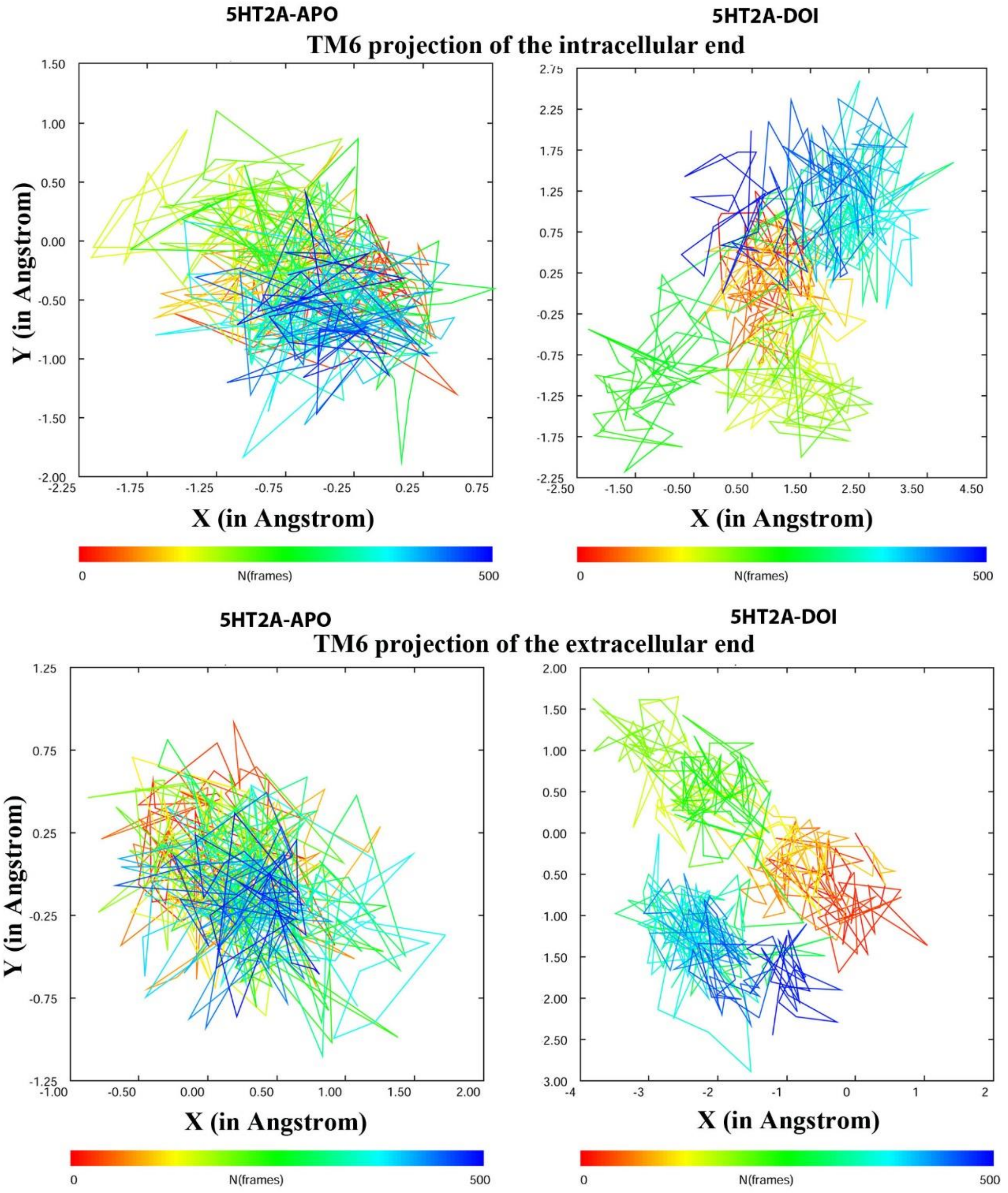

Figure S3. Eigenvectors (EVs) from Combined Principal Component Analysis (PCA) of 5HT2A-APO/DOI based on the MD simulations (200 – 1000ns, Cα atoms of the receptor). (A) 2D projection plot of the first two principal eigenvectors. (B) Plot of eigenvalues versus the corresponding eigenvector indices for the top 20 EVs. (C) Cumulative contributions of top 20 EVs.

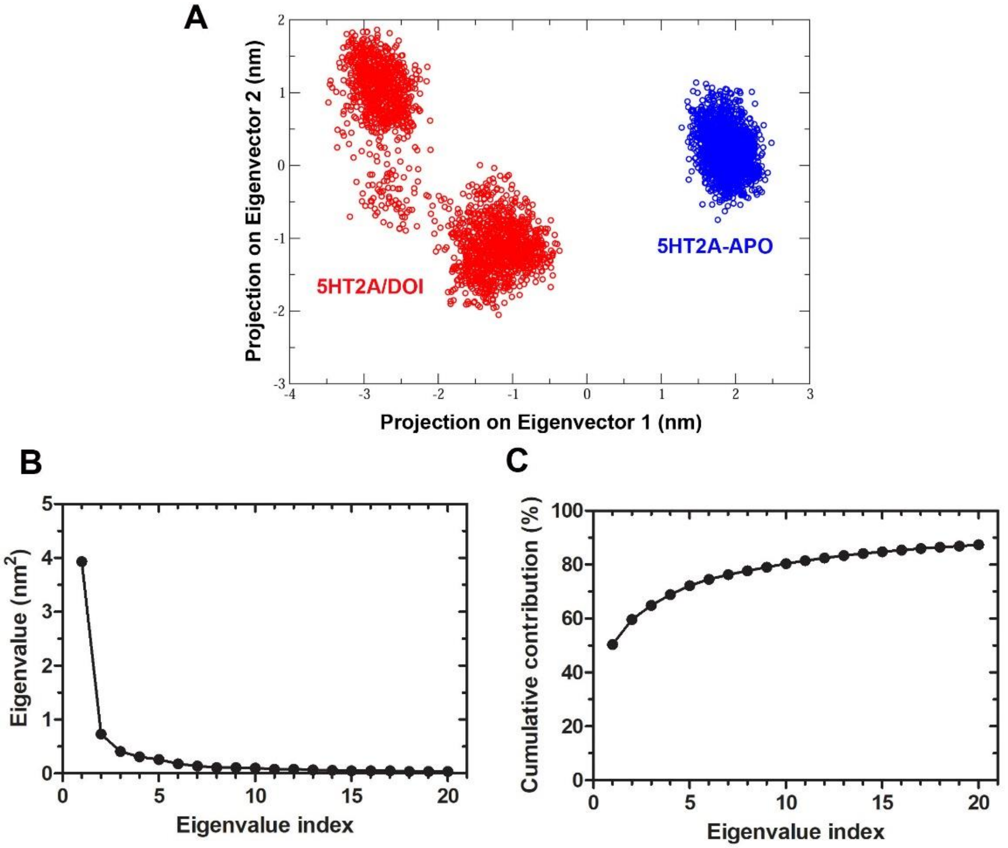

Table S1. The eigenvalues and percentage contribution of each eigenvector (top 20).

Eigenvector	Eigenvalue (nm)	Contribution (%)	Eigenvector	Eigenvalue (nm)	Contribution (%)
1	3.93	50.35%	11	0.08	1.08%
2	0.73	9.28%	12	0.08	0.99%
3	0.41	5.24%	13	0.07	0.92%
4	0.31	3.99%	14	0.06	0.79%
5	0.26	3.34%	15	0.05	0.65%
6	0.18	2.30%	16	0.05	0.59%
7	0.14	1.76%	17	0.05	0.58%
8	0.11	1.46%	18	0.04	0.49%
9	0.11	1.36%	19	0.04	0.46%
10	0.10	1.27%	20	0.04	0.45%

Figure S4. Dial plots for Proline Kink P246 (5.50) in TM5 for $5HT_{2A}$/APO, $5HT_{2A}$/GSK215803 and $5HT_{2A}$/DOI (200 – 1000ns). Average Bend, Wobble angles and Face shift are for APO (9.75, 46.85, 63.51), GSK215803 (11.62, 42.16, 59.66), and DOI (9.35, -98.11, 59.09), respectively.

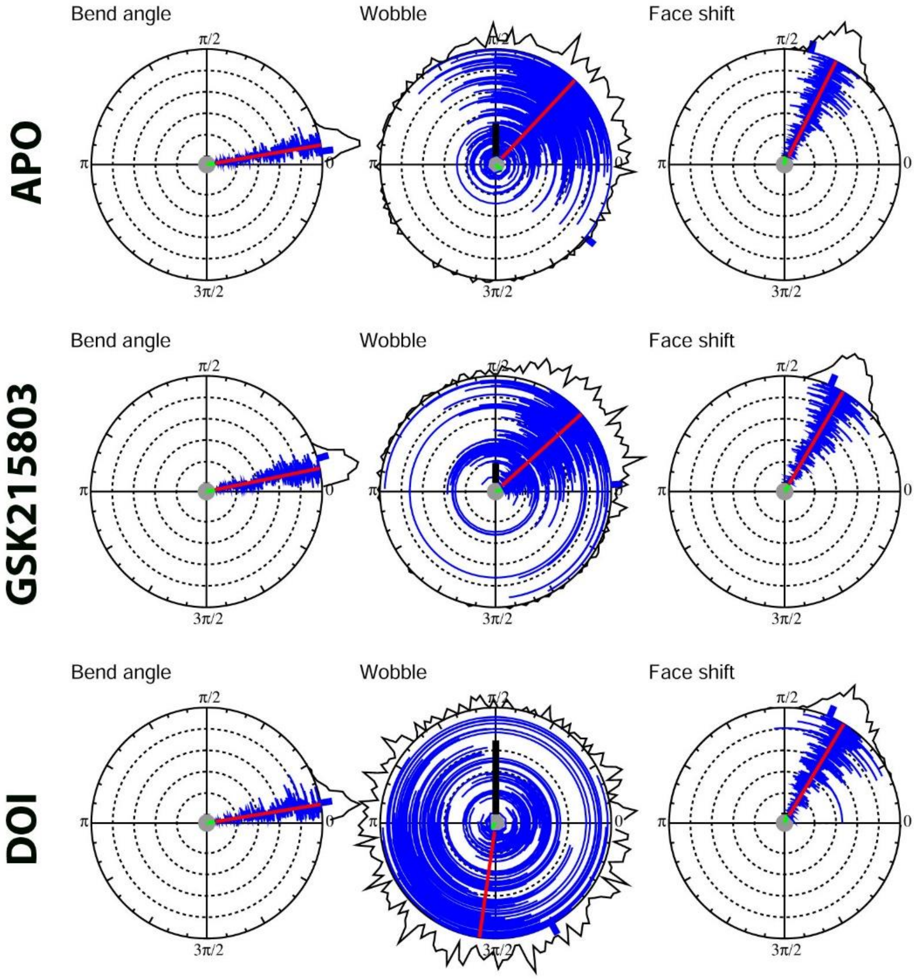

Figure S5. Dial plots for Proline Kink P338 (6.50) in TM6 for $5HT_{2A}$/APO, $5HT_{2A}$/GSK215803 and $5HT_{2A}$/DOI (200 – 1000ns). Average Bend, Wobble angles and Face shift are for APO (34.46, -63.11, 89.26), GSK215803 (34.68, -81.21,69.45), and DOI (34.51, -81.31, 56.68), respectively.

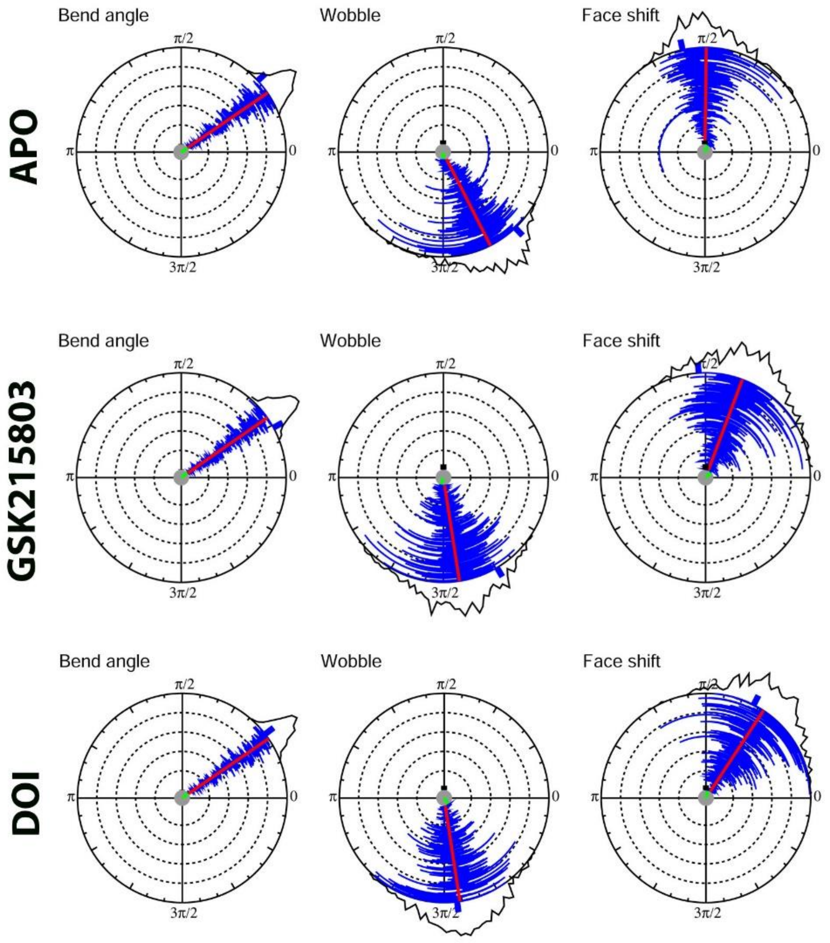

Figure S6. Dial plots for Proline Kink P377 (7.50) in TM7 for $5HT_{2A}$/APO, $5HT_{2A}$/GSK215803 and $5HT_{2A}$/DOI (200 – 1000ns). Average Bend, Wobble angles and Face shift are for APO (28.54, -122.36, 54.55), GSK215803 (28.84, -121.53, 52.41), and DOI (39.90, -68.10, 73.95), respectively.

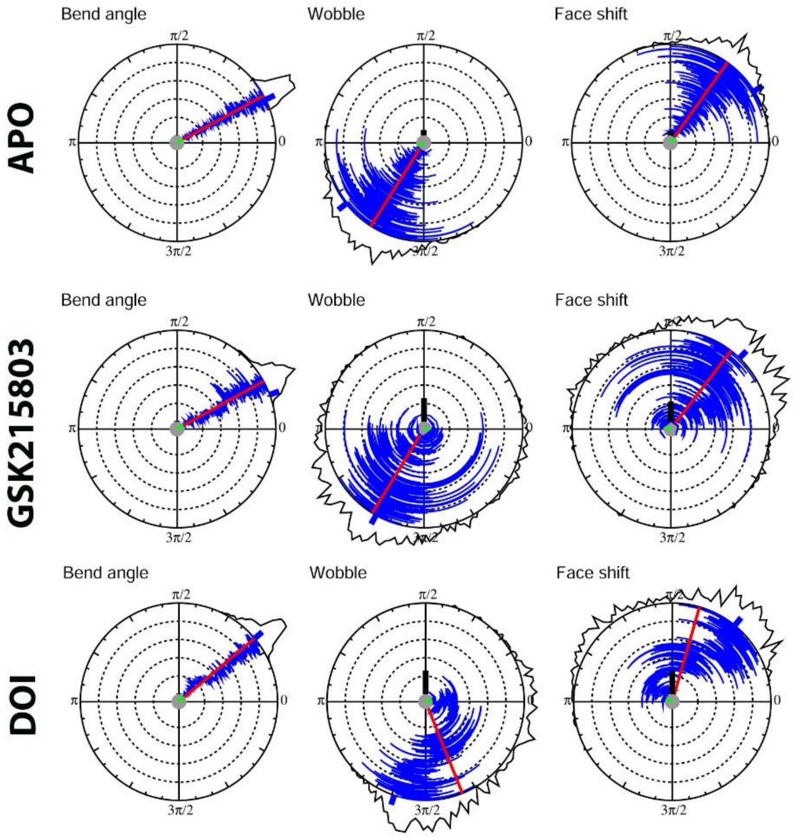

Figure S7. Dial plots for the Tryptophan Toggle Switch W6.48 in the TM6 for $5HT_{2A}$/APO, $5HT_{2A}$/GSK215803 and $5HT_{2A}$/DOI (200 – 1000ns). Average phi/psi angles are for APO (-106.26, -29.79), GSK215803 (-97.10, -21.52), and DOI (-90.82, -15.48). Average side chain torsional angles are for APO (-90.06, 113.48), GSK215803 (-83.91, 102.35), DOI (-79.26, 105.59).

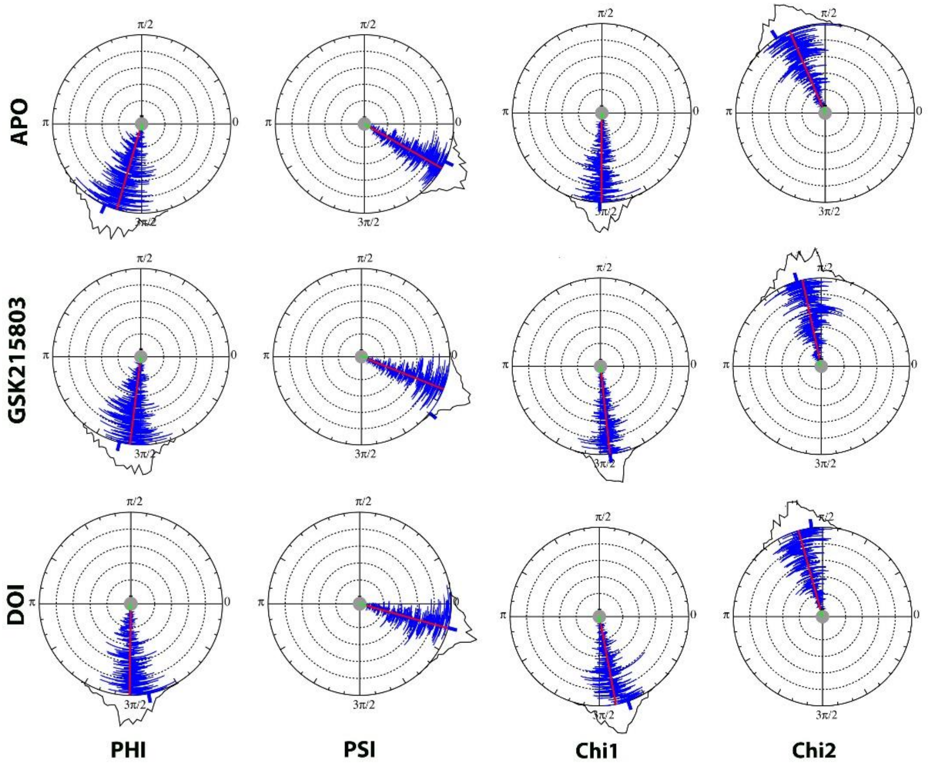

Figure S8. Dial plots for side chain torsional angles of F6.44 in the TM6 for $5HT_{2A}$/APO, $5HT_{2A}$/GSK215803 and $5HT_{2A}$/DOI (200 – 1000ns). Average side chain torsional angles are for APO (-63.52, 171.65), GSK215803 (168.54, 86.18), DOI (-146.62, 146.05).

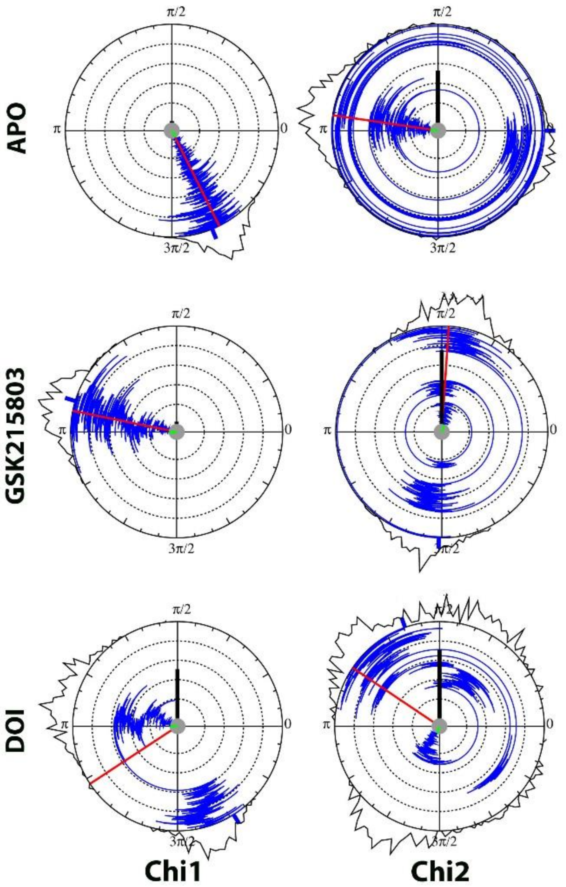

Figure S9. Dial plots of phi/psi angle distributions for residues G5.42 and S5.43 in the TM5 for $5HT_{2A}$/APO, $5HT_{2A}$/GSK215803 and $5HT_{2A}$/DOI (200 – 1000ns). Average phi/psi angles are for APO (-74.24/26.60; -156.82/-54.86), GSK215803 (-73.70/25.33; -155.16/-55.72), and DOI (-63.15/-42.33; -65.45/-33.30).

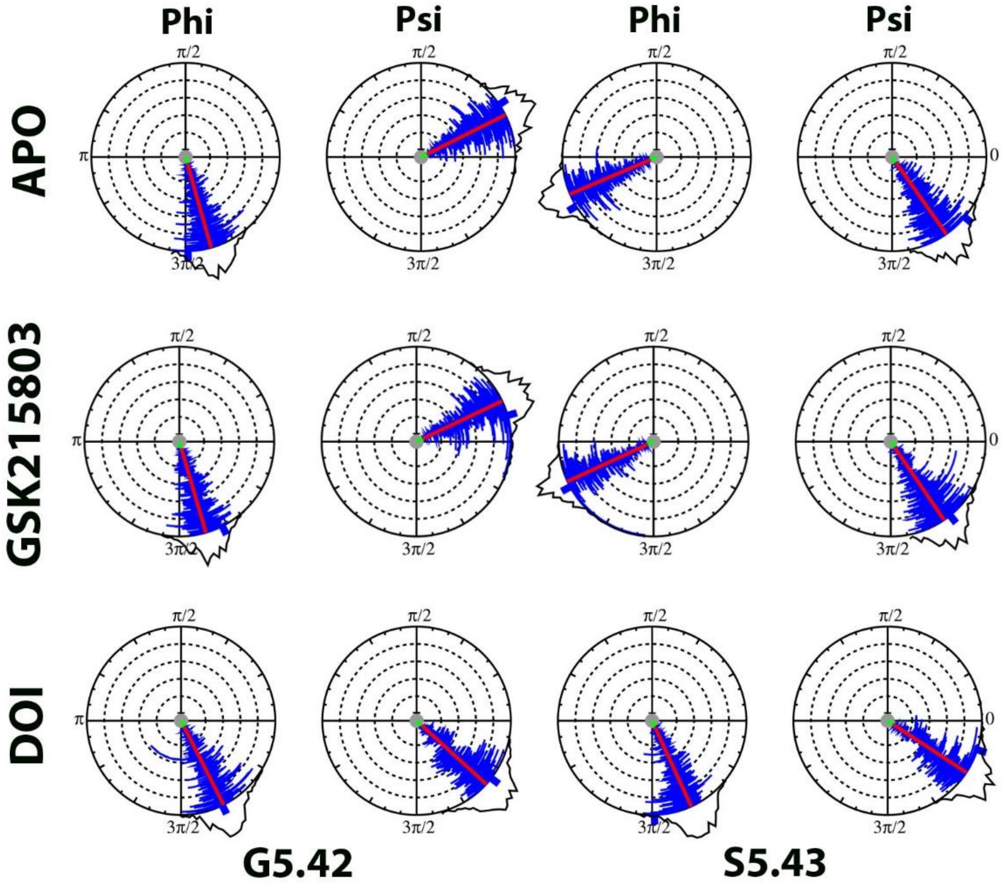

Figure S10. Selected salt bridge tracking during the MD simulations (200-1000ns) of $5HT_{2A}$/DOI (A) and $5HT_{2A}$/APO (B).

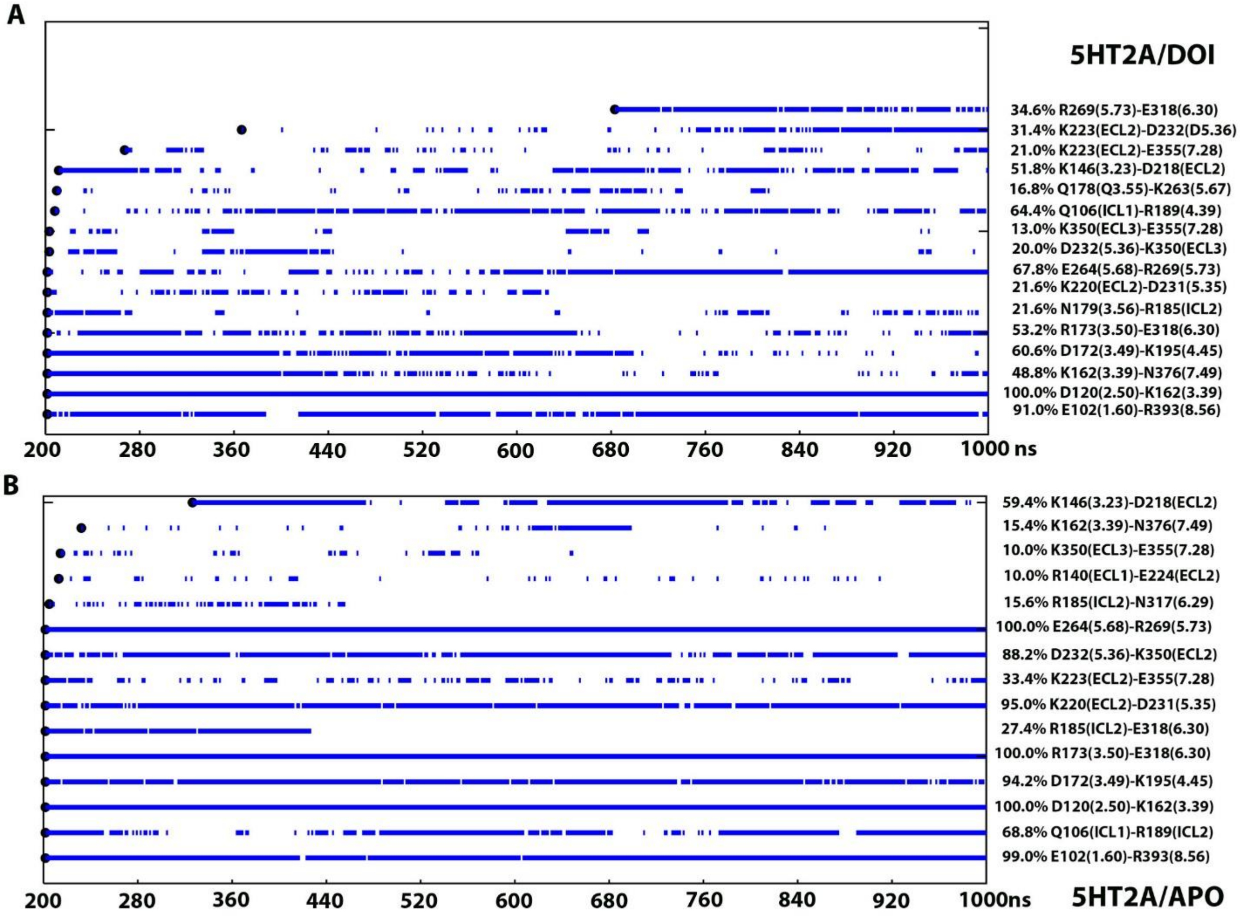

Figure S11. Heat maps for hydrophobic interaction difference between 5HT$_{2A}$/APO and 5HT$_{2A}$/DOI during MD simulations (200 – 1000 ns). Green arrows point to the residue pairs shown in the **Figure 8**.

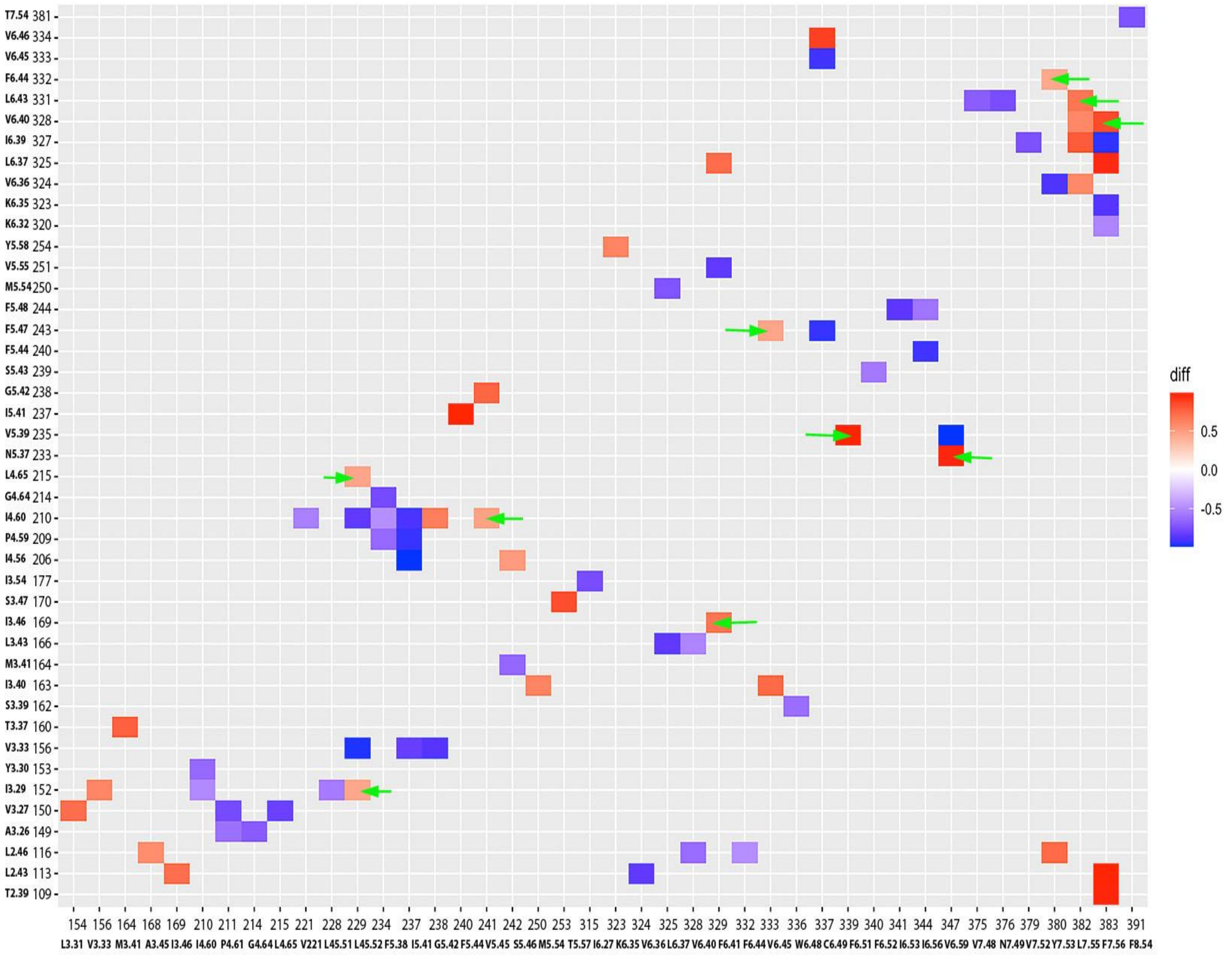

Figure S12. Heat maps for correlation pair difference between $5HT_{2A}$/APO and $5HT_{2A}$/DOI during MD simulations (200 – 1000 ns).

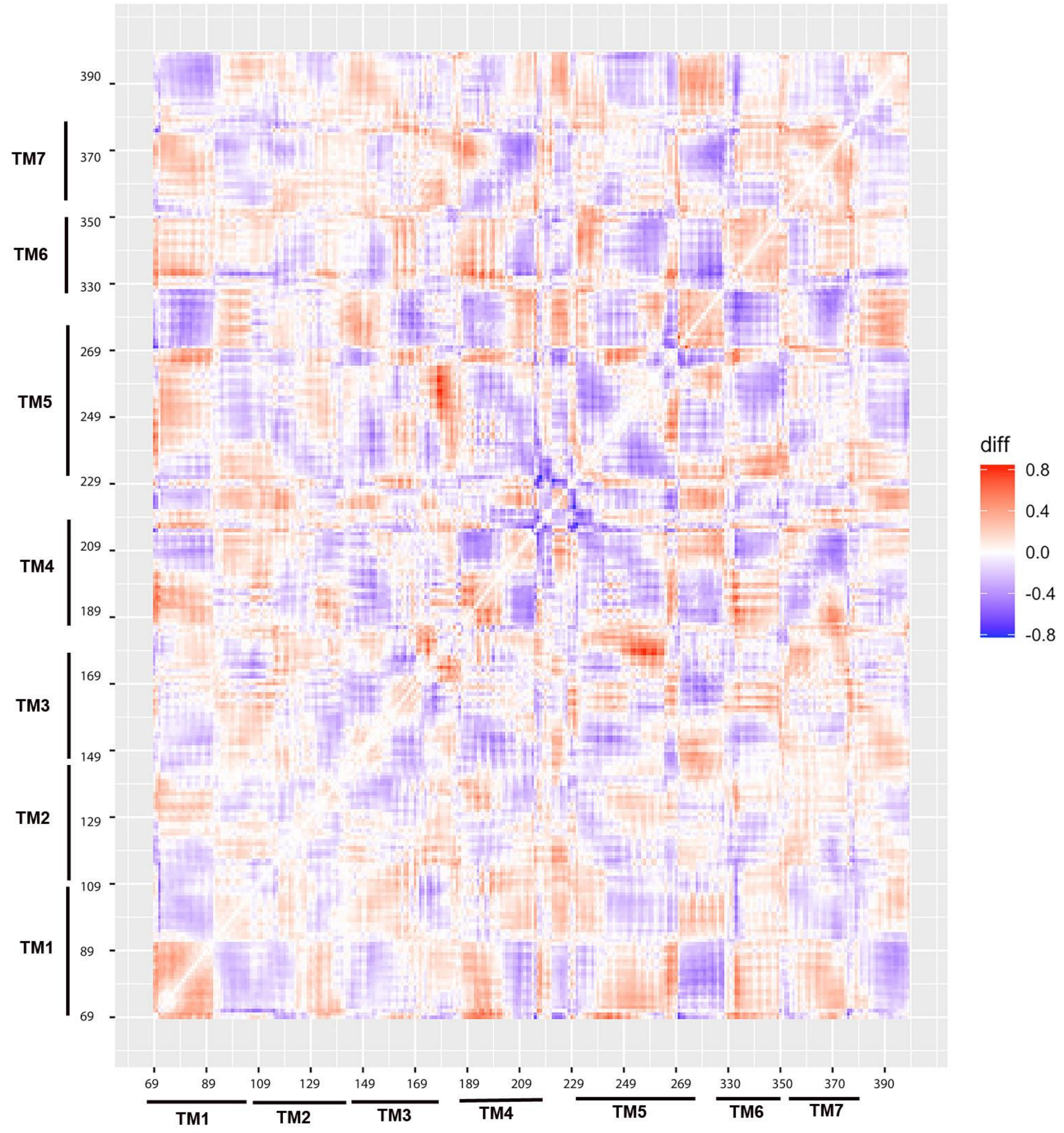

Table S2. Formed and broken salt bridge pairs in the $5HT_{2A}$/DOI system compared to the APO system from MD simulation trajectories (200 – 1000ns).

Broken		Formed	
	Diff. in Fraction		Diff. in Fraction
K350(ECL3)-D232(5.36)	-0.88	D232(5.36)- K223(ECL2)	0.31
D231(5.35)-K220(ECL2)	-0.73	E351(ECL3)-K223(ECL2)	0.21
E318(6.30)-R173(3.50)	-0.47		
K195(4.45)-D172(3.49)	-0.33		
E355(7.28)-K223(ECL2)	-0.31		

Table S3. Formed and broken hydrogen bond pairs in the $5HT_{2A}$/DOI system compared to the APO system from MD simulation trajectories (200 – 1000ns).

Broken		Formed	
	Diff. in Fraction		Diff. in Fraction
T253(5.57)-S170(3.47)	-0.87	N343(6.55)-D232(5.36)	0.89
V241(5.45)-W164(3.41)	-0.72	N376(7.49)-D120(2.50)	0.85
W151(3.28)-L126(2.56)	-0.65	W151(3.28)-V127(2.57)	0.73
		S207(4.57)-153(3.30)	0.51
		S242(5.46)-T160(3.37)	0.44
		W367(7.40)-S131(2.61)	0.30
		N376(7.49)-N92(1.50)	0.27
		S260(5.64)-Q178(3.55)	0.22
		Y380(7.53)-L113(2.43)	0.21

Table S4. Formed and broken hydrophobic interaction pairs in the $5HT_{2A}$/DOI system compared to the APO system from MD simulation trajectories (200 – 1000ns).

Broken		Formed	
	Diff. in Fraction		Dif. in Fraction
I237(5.41)-I206(I4.56)	-0.99	F240(5.44)-I237(5.41)	0.99
V347(6.59)-V235(5.39)	-0.99	F383(7.56)-L113(2.43)	0.98
L229(45.52)-V156(3.33)	-0.97	F339(6.51)-V235(5.39)	0.98
F383(7.56)-I327(6.39)	-0.95	F383(7.56)-T109(2.39)	0.96
C337(6.49)-F243(5.47)	-0.94	V347(6.59)-N233(5.37)	0.95
I237(5.41)-P209(4.59)	-0.94	V347(5.51)-N233(5.37)	0.95
C337(6.49)-V333(6.45)	-0.92	F383(7.56)-L325(6.37)	0.94
I344(6.56)-F240(5.44)	-0.92	C337(5.41)-V334(6.46)	0.88
I237(5.41)-I210(4.60)	-0.92	F383(7.56)-V328(6.40)	0.85
Y380(7.53)-V324(6.36)	-0.89	T253(5.57)-S170(3.47)	0.84
G238(5.42)-V156(3.33)	-0.87	L382(7.55)-I327(6.39)	0.79
F383(7.56)-K323(6.35)	-0.87	I163(3.40)-T160(3.37)	0.77
I341(6.53)-F244(5.48)	-0.85	V241(5.45)-G238(5.42)	0.75
L229(45.52)-I210(4.60)	-0.84	V333(6.45)-I163(3.40)	0.73
L325(6.37)-L166(3.43)	-0.84	Y380(7.53)-L116(2.46)	0.73
F329(6.41)-V251(5.55)	-0.84	F329(6.41)-L325(6.37)	0.72
V324(6.36)-L113(2.43)	-0.84	L154(3.31)-V150(3.27)	0.71
I237(5.41)-V156(3.33)	-0.82	I169(3.46)-L113(2.43)	0.71
L215(4.65)-V150(3.27)	-0.81	F329(6.41)-I169(3.46)	0.68
F234(5.38)-G214(4.64)	-0.77	L382(7.55)-L331(6.43)	0.68
P211(4.61)-V150(3.27)	-0.77	G238(5.42)-I210(4.60)	0.64
N376(7.49)-L331(6.43)	-0.76	M250(5.54)-I163(3.40)	0.62
I315(6.27)-I177(3.54)	-0.76	K323(6.35)-Y254(5.58)	0.62
F329(6.41)-L247(5.51)	-0.74	V156(3.33)-I152(3.29)	0.61
F391(8.54)-T381(7.54)	-0.74	L382(7.55)-V328(6.40)	0.60
V379(7.52)-V327(6.39)	-0.74	L382(7.55)-V324(6.36)	0.59
L325(6.37)-M250(5.54)	-0.73	L166(3.43)-L116(2.46)	0.57
G214(4.64)-A149(3.26)	-0.70	S242(5.46)-I206(4.56)	0.51
V375(7.48)-L331(6.43)	-0.69	V241(5.45)-I210(4.60)	0.47
S242(5.46)-W164(3.41)	-0.65	V333(6.36)-F243(5.47)	0.46
F234(5.38)-P209(4.59)	-0.64	L229(45.52)-L215(4.65)	0.46
V328(6.40)-L116(2.46)	-0.63	L215(4.65)-I152(3.29)	0.45
I210(4.60)-Y153(Y3.30)	-0.64	Y380(7.53)-F332(F6.44)	0.44
W336(6.48)-K162(3.39)	-0.62		
P211(4.61)-A149(3.26)	-0.61		
I344(6.56)-F244(5.48)	-0.59		
F340(6.52)-S239(5.43)	-0.57		
L228(45.51)-I152(3.29)	-0.56		
Y380(7.53)-V328(6.40)	-0.54		
V221(ECL2)-I210(4.60)	-0.54		
F383(7.56)-K320(6.32)	-0.52		
V328(6.40)-L166(3.43)	-0.52		
I210(4.60)-I152(3.29)	-0.50		
F234(5.38)-I210(4.60)	-0.48		
F332(6.44)-L116(3.43)	-0.48		

Table S5. Anti-correlation and correlation pairs in the $5HT_{2A}$/DOI system compared to the APO system from MD simulation trajectories (200 – 1000ns).

Anti-correlation		correlation	
	Diff. in Fraction		Diff. in Fraction
D217(4.67x68)-V235(5.35x36)	-0.83	V333(6.45)-K191(4.41)	0.72
P209(4.59)-V333(6.45)	-0.57	I197(4.47)-S188(4.58)	0.60
G225-L267(5.71)	-0.56		
V333(6.45)-K323(6.35)	-0.64		
C167(3.44)-A321(6.33)	-0.55		

Figure S13. Ionic lock distances of the receptors as function of simulation time (ns), **A.** Molecular Dynamics Simulation (MDS) primary. **B.** MDS replicate 1. **C.** MDS replicate 2.

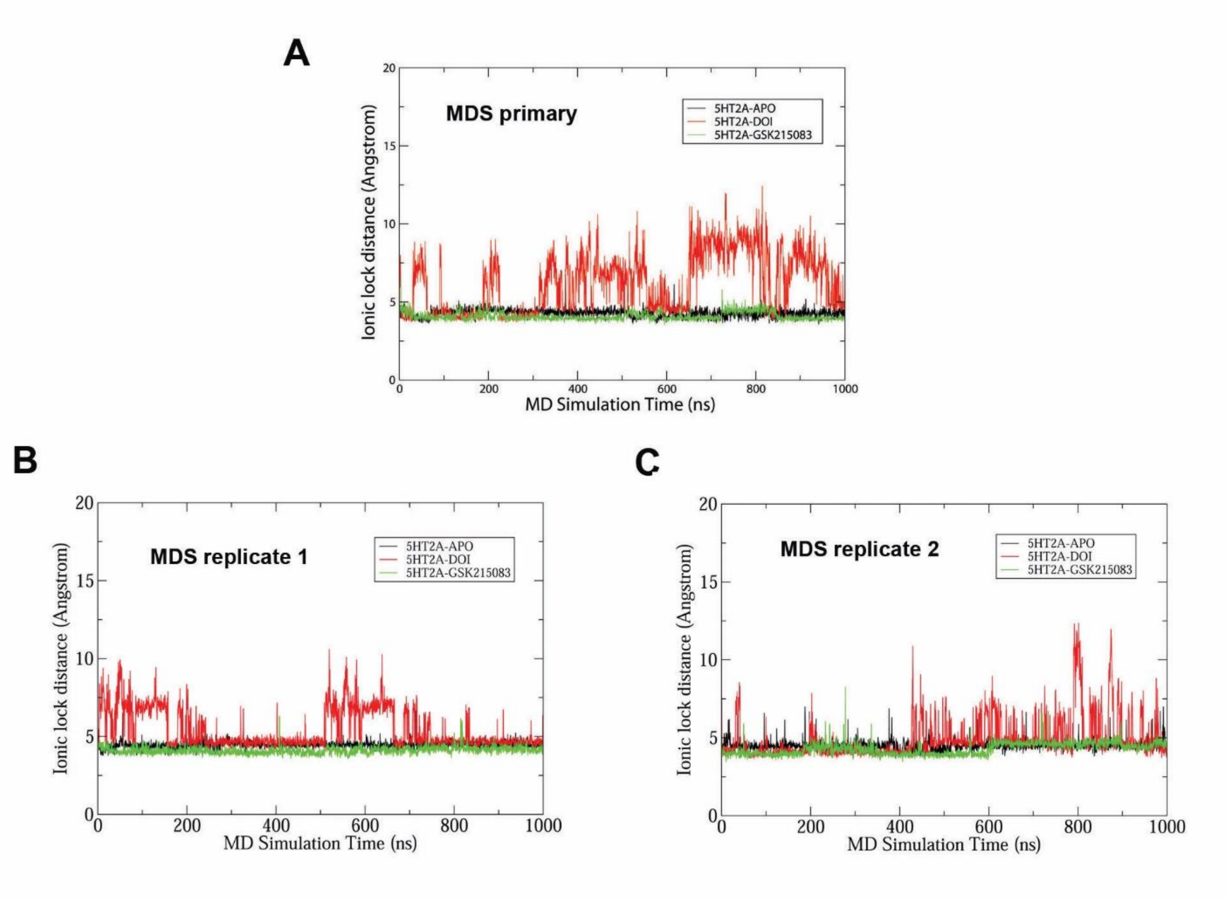

Supplement: Supplementary file 1 [file molecules-29-04935-s001.zip › molecules-3176095-supplementary/molecules-3176095-supplementary.pdf]
